# Analysis of the metabolomic profile in serum of irradiated nonhuman primates treated with Ex-Rad, a radiation countermeasure

**DOI:** 10.1038/s41598-021-91067-9

**Published:** 2021-06-01

**Authors:** Yaoxiang Li, Michael Girgis, Stephen Y. Wise, Oluseyi O. Fatanmi, Thomas M. Seed, Manoj Maniar, Amrita K. Cheema, Vijay K. Singh

**Affiliations:** 1 Department of Oncology, Lombardi Comprehensive Cancer Center, Georgetown University Medical Center, Washington, DC USA; 2 Division of Radioprotectants, Department of Pharmacology and Molecular Therapeutics, F. Edward Hébert School of Medicine, Uniformed Services University of the Health Sciences, 4301 Jones Bridge Road, Bethesda, MD 20814 USA; 3 Armed Forces Radiobiology Research Institute, Uniformed Services University of the Health Sciences, Bethesda, MD USA; 4 Tech Micro Services, 4417 Maple Avenue, Bethesda, MD 20814 USA; 5 Onconova Therapeutics, Inc, 375 Pheasant Run, Newtown, PA USA; 6 Department of Biochemistry, Molecular and Cellular Biology, Georgetown University Medical Center, Washington, DC USA

**Keywords:** Biomarkers, Diagnostic markers

## Abstract

To date, the United States Food and Drug Administration (FDA) has approved four drugs to mitigate hematopoietic acute radiation syndrome and all four are repurposed radiomitigators. There are several additional drug candidates currently under evaluation that may also be helpful for use during a widespread emergency. One possible candidate is Ex-Rad, also known as ON01210, a chlorobenzyl sulfone derivative (organosulfur compound), which is a novel, small-molecule kinase inhibitor with demonstrated efficacy in the murine model. In this study, we have evaluated the metabolomic and lipidomic profiles in serum samples of nonhuman primates (NHPs) treated with Ex-Rad after exposure to ionizing radiation. Two different dose administration schedules (Ex-Rad I administered 24 and 36 h post-irradiation, and Ex-Rad II administered 48 and 60 h post-irradiation), were used and evaluated using a global molecular profiling approach. We observed alterations in biochemical pathways relating to inflammation and oxidative stress after radiation exposure that were alleviated in animals that received Ex-Rad I or Ex-Rad II. The results from this study lend credence to the possible radiomitigative effects of this drug possibly via a dampening of metabolism-based tissue injury, thus aiding in recovery of vital, radiation-injured organ systems.

## Introduction

Although efforts to develop countermeasures for acute radiation syndrome were initiated more than 6 decades ago, only a limited number of effective and safe countermeasures for ARS gained approval by the United States Food and Drug Administration (US FDA) for radiation exposure scenarios^1–3^. These agents are repurposed drugs from their initial indications. However, these drugs, although approved, have their own safety concerns and risks. Hence, there is a constant need to develop additional, safe and effective medical countermeasures that could be kept in the strategic national stockpile^1–3^. These agents could be used to treat individuals that have been exposed or are at risk of injurious, ionizing radiation exposures following nuclear/radiological disasters and associated mass casualty events. The ideal treatment would be safe, effective, easily administered, able to be stockpiled for a long period of time at ambient conditions on a shelf in a warehouse, and would allow for minimal patient monitoring after administration^4^.

One candidate under development is Ex-Rad; a drug that has demonstrated significant protection against ^60^Co γ-irradiation when administered subcutaneously (*sc*, 500 mg/kg) to C3H/HeN mice 24 h and 15 min before irradiation, and has an estimated dose reduction factor of 1.16^5^. In another study, Ex-Rad demonstrated a significant survival benefit after prophylactic oral (*po*) administration^6^. Also, it was reported that Ex-Rad exerts its putative radioprotective efficacy by reducing the levels of pro-apoptosis proteins such as p53 and its downstream regulators p21, Bax, c-Abl, and p73, suggesting that Ex-Rad interferes with cellular damage that arises from ionizing radiation-induced p53-dependent apoptosis^5^. Furthermore, Ex-Rad treatment appears to significantly limit hematopoietic injury within acutely irradiated rodents^5,7^. Some studies suggested that Ex-Rad acts by blocking ATM and preventing accumulation of p53 after exposure; a process that serves to abrogate p-53 mediated programmed cell death^8^. Kang et al*.* demonstrated that Ex-Rad manifests its protective effects through the up-regulation of phosphatidylinositol 3-kinase (PI3K)/Akt (Serine/threonine kinase, protein kinase B) pathways in cells exposed in vitro to ionizing radiation^9^.

While we fully understand that the mechanism of action of Ex-Rad is essential for regulatory agency approval(s) (i.e., for FDA’s granting of the drug’s Investigational New Drug (IND) status), it is also crucial to contemplate and optimize effective treatment strategies^10^. With this in mind, we observed recently that Ex-RAD upregulates p-AKT2 in unirradiated as well as irradiated mice and NHPs when administered subcutaneously. We used bone marrow and peripheral blood mononuclear cells for p-AKT2 analysis and observed upregulation at various time points after drug injection in irradiated as well as unirradiated mice and NHPs (unpublished observation—manuscript under preparation). The latter observations are consistent with a report that AKT1 and AKT2 assist in the maintenance hematopoietic stem cell function by regulating reactive oxygen species^11^. We examined the effect of time on drug effectiveness by treating irradiated animals with two doses of the test drug at two distinct time frames following radiation exposure (one drug regimen at 24 and 36 h post-irradiation and another regimen at 48 and 60 h post-irradiation).

In this study, NHPs were exposed to single doses of ionizing radiation (7.2 Gy, LD_70/60_). Serum samples were obtained from three different treatment groups: Ex-Rad I administered 24 and 36 h post-irradiation, Ex-Rad II administered 48 and 60 h post-irradiation, and a vehicle-treated group. Samples were collected 4 days prior to irradiation (SD-4), 1 day (SD1), and 4 days (SD4) after irradiation in order to monitor the efficacy of each treatment and to perform untargeted metabolomic and lipidomic profiling. SD1 sample was collected just prior to Ex-Rad administration to Ex-Rad I group. Multivariate analyses of these data helped demonstrate that the administration of Ex-Rad leads to specific blood based metabolic signatures; signatures that might suggest tentatively, a degree of restorative benefit from acute radiation injury. This study also demonstrates the utility of metabolomics in determining basic, underlying processes involved in the radioprotective efficacy of Ex-Rad. This analysis may aid in the identification of reliable biomarkers for radioprotective efficacy of other countermeasures under development.

## Results

**Figure 1:**
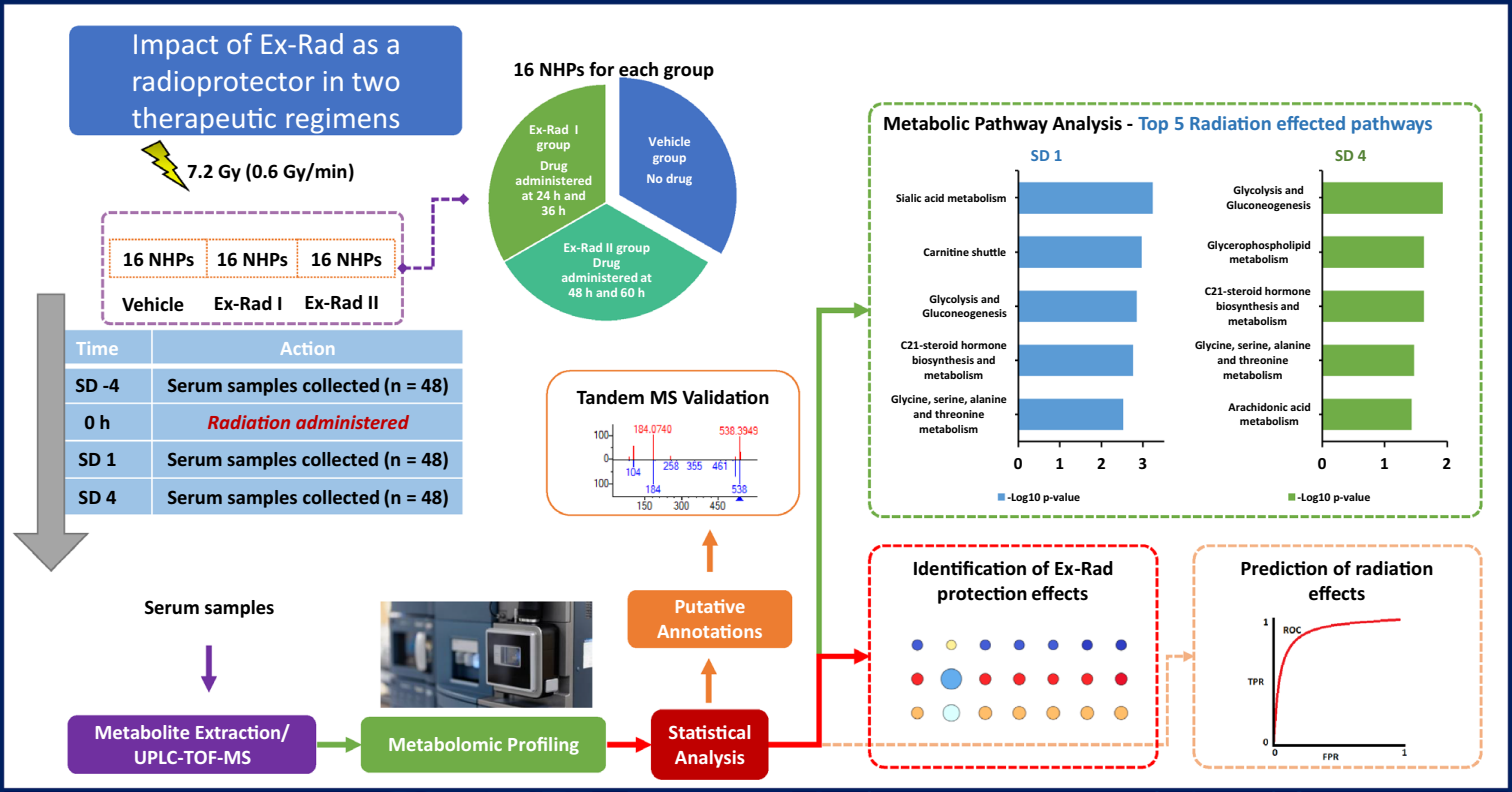
Overall experimental design for biomarker discovery of radiation exposure and Ex-Rad I and II treatments. Figure is created by the author using Microsoft PowerPoint (URL: https://www.microsoft.com/en-us/microsoft-365/powerpoint).

Ex-Rad is a potential radiation countermeasure^5,7,12^. However, no clear treatment strategy is yet inferred from the literature. Since the onset of treatment is crucial in any proposed method of treatment especially those directed towards ARS, we examined two potentially optimal time frames that would allow the test drug to provide optimal therapeutic efficacy. We allocated NHPs in 3 different groups and exposed them to a single dose of 7.2 Gy of ionizing radiation. Group I received vehicle (administered 24 and 36 h post-irradiation), group II received Ex-Rad at 24 and 36 h post-irradiation, and group III received Ex-Rad administered 48 and 60 h post-irradiation. Serum samples were collected and analyzed utilizing high resolution mass spectrometry-based untargeted metabolomics and lipidomics profiling methods (Fig. 1).

Lipidomic data were pre-processed using XCMS; 703 and 912 features were detected in the electrospray positive and negative modes, respectively. Pre-processing of metabolomic LC–MS data revealed that 2,245 and 2,133 features were detected in the electrospray positive and negative modes, respectively. In addition, we performed a tandem MS/MS validation test to verify those statistically significant features. A total of 139 annotated metabolites were significantly dysregulated (Supplementary Table 1).

### Ionizing radiation caused metabolic perturbation in serum of NHP at SD1 and SD4

**Figure 2:**
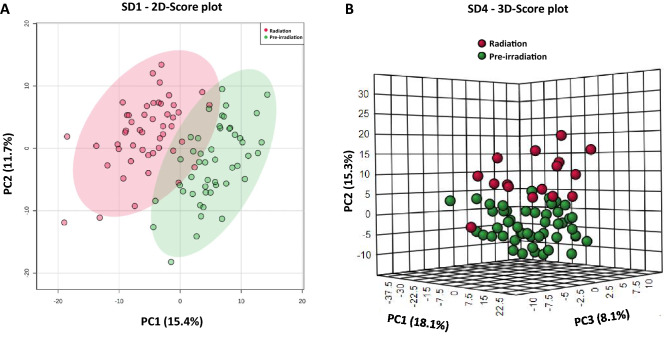
Exposure to ^60^Co gamma-radiation induces short term changes in serum metabolic profiles in NHPs. Panel A. First two-principal components two dimensional score plot for NHPs at SD1. Panel B. First three-principal components 3D score plot for NHPs at SD4. Figure is created by open source software MetaboAnalyst (version 5.0, URL: https://www.metaboanalyst.ca).

**Figure 3:**
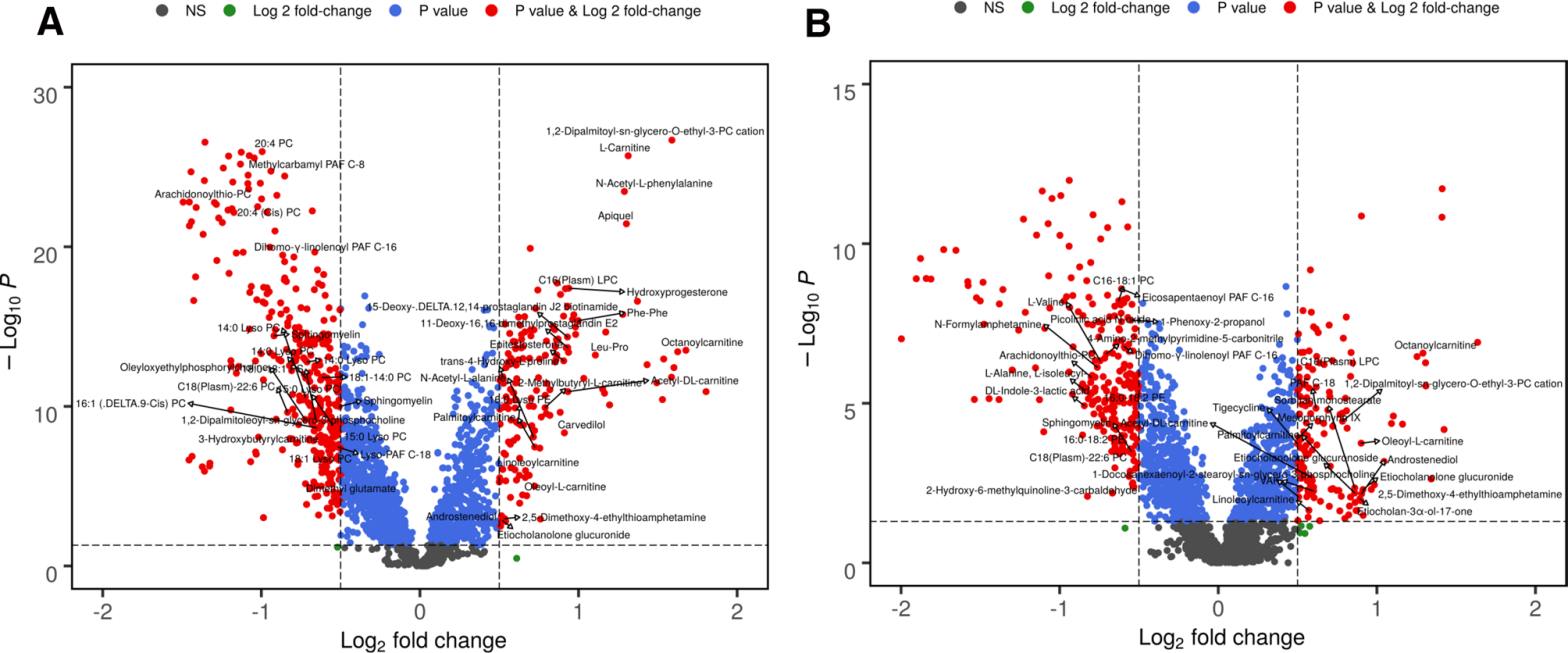
Volcano plot showing comparison of NHP serum profiles at SD1 (**A**) and SD4 (**B**). All annotated metabolites were validated by tandem mass spectrometry and have a significant FDR adjusted p value (< 0.05) comparing pre-irradiation vs. radiation. Figure is created by open source software R (version 4.0.3, URL: https://cran.r-project.org/).

Radiation effects were observed via metabolite fold change of features at SD1 and SD4 compared to the pre-irradiation group independently (Supplementary Table 2). A principal component analysis (PCA) demonstrated group differences between the radiation and pre-irradiation group (Fig. 2). While Fig. 2A demonstrates a 2D-score plot for SD1 data compared to pre-irradiation, Fig. 2B illustrates a 3D score plot of SD4 compared to pre-irradiation. To illustrate the overall pattern of the annotated metabolites, volcano plots were constructed based on peak intensities of each metabolite compared to pre-irradiation for SD1 and SD4, respectively (Fig. 3, panels A and B). These plots disclose the important role played by carnitine shuttle related metabolites upon exposure to ionizing radiation at SD1 and SD4. Out of all annotated metabolites, 77 were significantly dysregulated for both SD1 and SD4. Only 2 metabolites were uniquely dysregulated within SD1 samples, while 33 were found to be significantly different, particularly within SD4 samples (Supplementary Table 3).

In an effort to understand the action of ionizing radiation mechanistically, all detected features were incorporated in a comprehensive pathways analysis utilizing Mummichog 2.06 software (URL: http://mummichog.org/) (Supplementary Table 4)^13^. The outcome of this analysis revealed that several pathways were significantly dysregulated in both the SD1 and SD4 samples including Sialic acid metabolism, carnitine shuttle, glycine, serine, alanine and threonine metabolism, glycerophospholipid metabolism, tyrosine metabolism, urea cycle/amino group metabolism, glycosphingolipid biosynthesis—globoseries, galactose metabolism, porphyrin metabolism as well as androgen and estrogen biosynthesis and metabolism. While sialic acid metabolism and carnitine shuttle pathways were heavily involved in SD1, pathways like limonene and pinene degradation, and fatty acid metabolism were only unique to SD4.

### Treatment with Ex-Rad partially reversed irradiation-induced metabolomic changes

**Figure 4:**
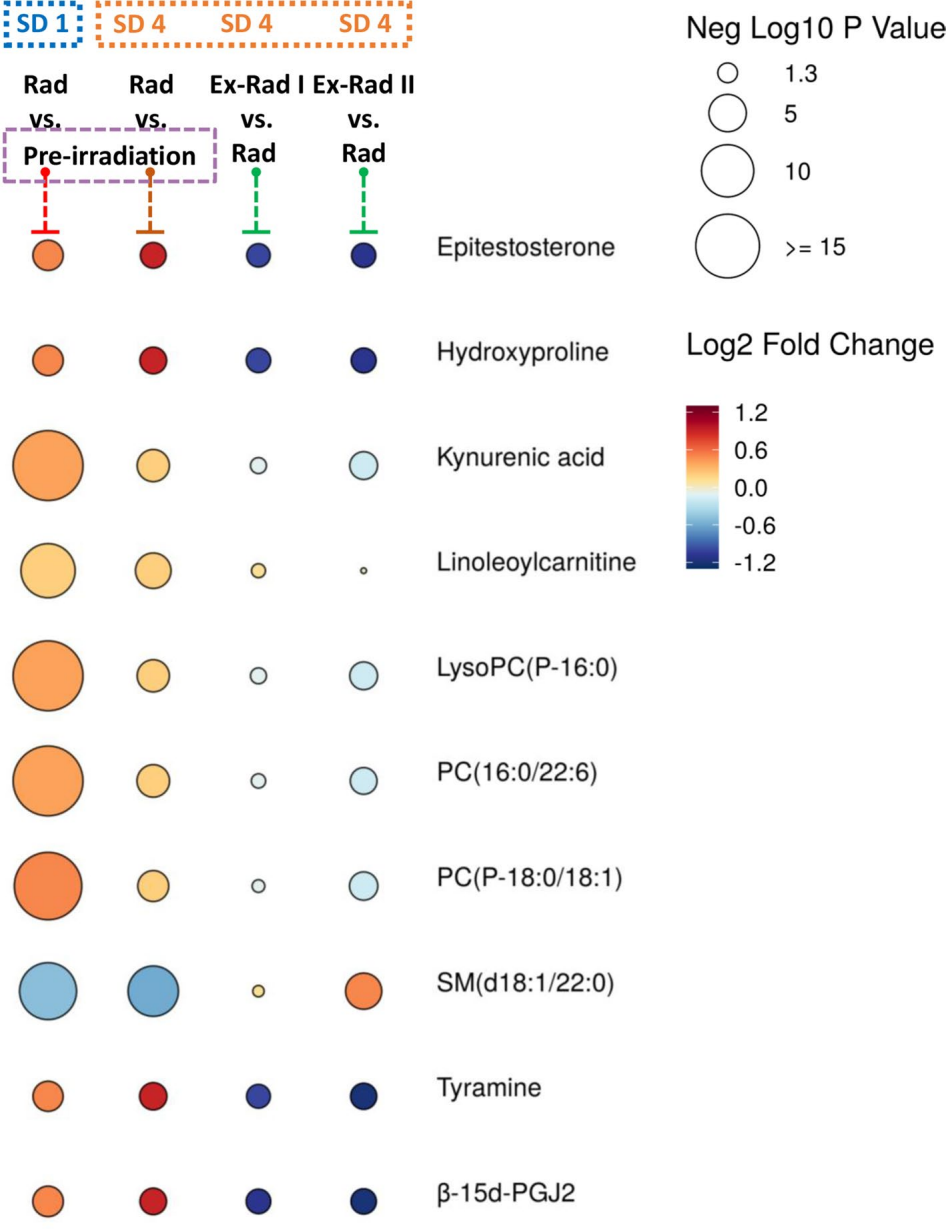
Raindrop plot illustration showing significantly altered metabolites by radiation in SD1, SD4, and then protected by Ex-Rad. Figure is created by open source software R (version 4.0.3, URL: https://cran.r-project.org/).

Next we interrogated the efficacy of Ex-Rad dosing regimen by comparing the vehicle group with Ex-Rad I and Ex-Rad II at SD4 (Supplementary Table 2). Multivariate analysis showed that 66 annotated metabolites reverted back to near normal abundance with the Ex-Rad I treatment approach, while a total of 83 metabolites showed a similar reversal effect using the Ex-Rad II treatment schedule. Several dysregulated metabolites such as phenylalanine, *N*-phenylacetylphenylalanine, Phe-Phe, and tyramine appear to have recovered using both treatments, whereas some metabolites such as β-15d-PGJ2, 11-deoxy-16,16-dimethyl PGE2, and octanoylcarnitine were only recovered in the Ex-Rad II treatment schedule. The raindrop plot in Fig. 4 also illustrates that several metabolites were dysregulated by irradiation at SD1, with the degree of dysregulation worsening at SD4, but apparently better regulated by the Ex-Rad treatments.

Mummichog pathway analysis (URL: http://mummichog.org/) (Supplementary Table 4) indicated that C21-steroid hormone biosynthesis and metabolism, porphyrin metabolism, and butanoate metabolism pathways were initially dysregulated by the acute irradiation, but then more normally regulated with both Ex-Rad I and Ex-Rad II treatment schedules. Surprisingly, glycine, serine, alanine and threonine metabolism, glycerophospholipid metabolism, tyrosine metabolism, and urea cycle/amino group metabolism were dysregulated by irradiation, but seemingly only better, more normally regulated using the Ex-Rad II treatment schedule.

Pathway analysis results (Supplementary Table 4) summarize the pathways that were significantly enriched in our metabolite dataset. We identified 13 metabolites affected in the carnitine shuttle pathway and 24 metabolites involved in the C21-steroid hormone biosynthesis and metabolism pathway.

### Machine learning based prediction model augments the prediction of short term radiation effects for NHPs

**Figure 5:**
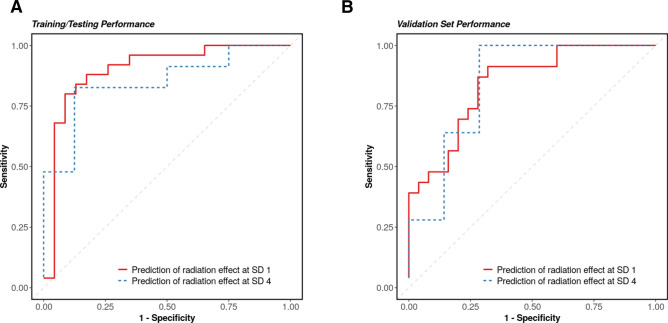
The biomarkers panel to distinguish whether NHP got radiation or not in SD 1 and SD 4. Four metabolites for SD 1 and SD 4 prediction: l-carnitine, PC(14:0/18:0), 2-thio PAF, 4-deoxyuridine. (**A**) training and testing performance yielded AUC = 0.899 for SD 1 model and AUC = 0.848 for SD 4 model. (**B**) validation performance with a AUC = 0.842 for SD 1 model and AUC = 0.846 for SD 4 model. Figure is created by open source software R (version 4.0.3, URL: https://cran.r-project.org/).

To predict acute radiation effects, we aimed to develop a high accuracy classification algorithm using LASSO regression at SD1 and SD4. For SD1, the prediction model contains four metabolites including l-carnitine, 20:4 PC, methylcarbamyl PAF C-8, and *N*-acetyl-l-phenylalanine. For SD4, the prediction model contains five metabolites including picolinic acid *N*-oxide, 1-phenoxy-2-propanol, C16-18:1 PC, octanoylcarnitine, and eicosapentaenoyl PAF C-16. Figure 5A indicates the training and the testing performance yield AUC was 0.991 for SD1 and 0.957 for SD4. Panel B, which is validation set performance, shows that SD1 has an AUC of 0.918 and SD4 has an AUC of 0.851.

## Discussion

For any given radiation medical countermeasure under study, it is essential that corresponding treatment strategies be developed in order to maximize a test drug’s effectiveness and utility. In this study, we studied two treatment regimens for Ex-Rad, a potential medical countermeasure against the injurious effects of acute IR. The only difference between the two treatments was the time frame in which the doses of the drug were administered. Ex-Rad I was administered 24 and 36 h post-irradiation, while Ex-Rad II was administered 48 and 60 h post-irradiation. There were 12 h differences between both the first and second dose of each of the treatment strategies. The goal of this study was to find the most effective treatment regimen relative to noted metabolic disturbances induced by ionizing radiation exposures. Our results clearly reveal that the two treatment strategies differed significantly.

As expected, both treatment strategies reversed a broad range of apparent, radiation induced alterations of serum metabolites. Furthermore, pathway analysis conveyed that several pathways were impacted by both treatments. For example, carnitine shuttle, C21-steroid hormone biosynthesis and metabolism, linoleate metabolism, leukotriene metabolism, androgen and estrogen biosynthesis and metabolism, prostaglandin formation from arachidonate, glutathione metabolism, and purine metabolism were influenced by both treatments.

Several metabolites were dysregulated by exposure to ionizing radiation but appeared to have recovered to various degrees following drug administration, especially at SD4. Carnitine shuttle is a crucial mechanism by which long chain fatty acids can enter through the impermeable mitochondrial membrane for the purpose of oxidation and energy production^14^. Acetylcholine, palmitoylcarnitine, octanoylcarnitine, linoleoylcarnitine, and oleoyl-l-carnitine were elevated initially in sera after irradiation, but these levels fell back at SD4 under both treatment schedules. This is evidence of the involvement of carnitine shuttle in the recovery process of select metabolites. Androstenediol and epitestosterone are good examples of C21-steroid hormone^15^. These hormones were elevated by ionizing radiation but returned to near normal levels under both treatment schedules; the Ex-Rad II schedule exhibited slightly better efficacy than did the Ex-Rad I schedule.

Tryptophan metabolites like l-kynurenine and kynurenic acid have shown similar patterns. The compound l-kynurenine is a metabolite of l-tryptophan. Kynurenine and its breakdown products carry out diverse biological functions, including dilating blood vessels during inflammation and modulating the immune response^16,17^. Kynurenic acid, a crucial metabolite of l-tryptophan, exerts its neuroprotective capabilities by blocking excitatory amino acid receptors^18^ and a ligand for orphan G protein-coupled receptor GPR35^19^ that plays an essential role in the inflammatory response. Again, both Ex-Rad treatments showed that they can lower the elevated levels of these metabolites, although slightly better results were noted using the Ex-Rad II treatment schedule.

Prostaglandine β-15d-PGJ2, a natural peroxisome proliferator-activated receptor-γ (PPAR-γ) agonist, plays many roles and exerts anti-tumor, anti-inflammatory, antioxidation, antifibrosis, and antiangiogenesis effects^20,21^. The radiation induced elevated levels of β-15d-PGJ2 were alleviated by both treatments. However, the Ex-Rad II schedule yielded results that were more statistically significant than the Ex-Rad I schedule.

In this study, we utilized machine learning (training and testing) computational tools to develop a prediction model that can anticipate and assess the efficacy of any therapeutic regimen that may utilize Ex-Rad either singly or in combination with other therapeutics. The best performing of these models were associated with metabolites l-carnitine, PC(14:0/18:0), 2-Thio PAF, and 4-deoxyuridine for SD1 and SD4 prediction. The AUC values were 0.899 for the SD1 model and 0.848 for SD4 model in training and testing set, while AUC = 0.842 for the SD1 model and AUC = 0.846 for the SD4 model in the validation set.

In summary, our findings underscore the metabolic benefit of Ex-Rad; as such, this ‘benefit’ might well serve to radiation injury, regardless of the two treatment schedules. However, the Ex-Rad II schedule might prove to be more efficient in quenching late inflammatory reactions. Further research is needed to target these inflammatory mediators modulated by Ex-Rad II for a better understanding of the mechanistic differences. Additional studies are also needed to provide better definition to both temporal and casual relationships between the mitigating, metabolic actions of the Ex-Rad drug and the subsequent repair and recovery of organ systems at risk to injury arising from acute IR exposures.

## Materials and methods

### Animals

Forty-eight naïve rhesus macaques (*Macaca mulatta*, Chinese substrain, all males) 4–5 years of age, weighing 4.3–6.2 kg, were obtained from the National Institutes of Health Animal Center (NIHAC, Poolesville, MD, USA) and were maintained in a facility accredited by the Association for Assessment and Accreditation of Laboratory Animal Care (AAALAC)-International. Animals were quarantined for 6 weeks prior to study initiation. Animal housing, health monitoring, care, and enrichment during the experimental period have been described earlier^22^. Animals were fed primate diet (Teklad T.2050 diet; Harlan Laboratories Inc., Madison, WI, USA) twice daily with at least 6 h between feedings (four biscuits each at 07:00 AM and 02:00 PM), received drinking water *ad libitum*, and also received daily nutritional enrichment. Due to study-specific reasons, paired housing was not possible during the experiment. The animals were housed individually, but were able to see and touch neighboring animals through the cage divider. Single housing also eliminated the chance of conflict injuries that could have been caused by pair-housing. Irradiated animals are more prone to infection due to their suppressed immune system. All procedures involving animals were approved (Protocol # P2013-12-016 approved on March 12, 2014) by the Institutional Animal Care and Use Committee (IACUC, Armed Forces Radiobiology Research Institute) and the Department of Defense Animal Care and Use Review Office (ACURO). This study was carried out in strict accordance with the recommendations in the *Guide for the Care and Use of Laboratory Animals* of the National Research Council of the National Academy of Sciences^23^. This study was carried out in compliance with the ARRIVE guideline.

### Experimental design

There were three treatment groups (16 NHPs per group) for this metabolomics study. Group I received vehicle (administered 24 and 36 h post-irradiation), group II received Ex-Rad 40 mg/kg *sc* at 24 and 36 h post-irradiation, and group III received 40 mg/kg of Ex-Rad administered 48 and 60 h post-irradiation. All animals were exposed to 7.2 Gy ^60^Co gamma-radiation, a radiation dose capable of inducing H-ARS and mild GI-ARS. Serum samples for metabolomic analysis were collected on SD -4, SD1, and SD4 (48 samples for each time point). A total of 144 serum samples were analyzed for their metabolomic profiles.

### Drug preparation and administration

Ex-Rad (ON01210, Recilisib, a chlorobenzylsulfone derivative—Onconova Therapeutics, Inc., Newtown, PA) was supplied in the form of a certified solution of 50 mg/ml in 0.2 M Tris-ethylenediaminetetraacetic acid (Tris–EDTA) buffer combined with PEG-400 at a 50:50 ratio. The receipt and storage of the test and control articles were handled under Good Laboratory Practice (GLP) conditions. Each vial was wrapped in aluminum foil to prevent exposure to light and stored in a locked cabinet within a temperature controlled room (14–28 °C) until time of use, as per the manufacturer’s instructions. The storage temperature was measured, verified, and recorded daily. The vehicle was also supplied in the form of a certified solution of 0.2 M Tris–EDTA buffer (Sigma-Aldrich, St. Louis, MO) containing 1% TPGS (d-α-tocopheryl polyethylene glycol succinate) (Sigma-Aldrich, St. Louis, MO) combined with PEG-400 (Sigma-Aldrich, St. Louis, MO) at a 50:50 ratio, and was handled and stored as described above. The irradiated NHPs were injected according to the assigned treatment regimen. The quantity of drug or vehicle injected was calculated on an individual basis based on body weight at SD0 to ensure a consistent 40 mg/kg dose. The area surrounding the injection site was shaved at least 48 h before drug administration so the site could be easily observed for any adverse skin reactions such as rash, inflammation, irritation, or abscess formation following Ex-Rad or vehicle administration. Immediately prior to drug injection, the injection site (dorsal scapular region—between the shoulder blades) was wiped with 70% isopropyl alcohol and allowed to air dry. NHPs were dosed *sc* with Ex-Rad or vehicle using a sterile 21–24-gauge needle attached to a 3–6 ml disposable luer-lock syringe.

### Radiation exposure

Animals were irradiated and dose rate measurements were performed as previously described^22,24,25^. In brief, after arriving at the Cobalt Facility, animals were sedated with ketamine hydrochloride [10–15 mg/kg intramuscular (*im*)] to limit movement while undergoing irradiation. Two NHPs, selected based on abdominal width pairing (± 1 cm), were secured in a restraint device and placed on the irradiation platform facing away from each other. The animals were exposed with a total-body midline dose of 7.2 Gy ^60^Co gamma-radiation at a rate of 0.6 Gy/min. To deliver the precise dose, NHPs’ abdominal widths were measured with digital calipers. The NHPs were continuously observed while undergoing the procedure using a real-time video monitoring system. After irradiation, the animals were transported back to the vivarium in the same manner as they arrived to recover from sedation. The animals were constantly monitored during their recovery time.

Dosimetry was based primarily on the alanine/EPR (electron paramagnetic resonance) system^25,26^, currently accepted as one of the most accurate methods for relatively high radiation doses, and used for comparisons between national metrology institutions. The calibration curves (EMXmicro spectrometer, Bruker Corp., Billerica, MA, USA) used in dose measurements at the Armed Forces Radiobiology Research Institute (AFRRI) are based on standard alanine calibration sets purchased from the US National Institute of Standards and Technology (NIST, Gaithersburg, MD, USA). The alanine dosimeters obtained from NIST had been calibrated in terms of absorbed dose to water using the US National Standard Radiation Sources. At AFRRI, identical alanine dosimeters were placed midline within NHP phantoms (Plexiglas cylinders 6.9, 10, 12.5 cm in diameter and 34.5 cm length) and irradiated to approximately 100 Gy. Measurement of their EPR signals using the calibration curve constructed with alanine dosimeters from NIST provided dose rates to water in the core bodies of NHPs. A small correction was subsequently applied for the difference in mass energy absorption coefficients between water and soft tissue.

### Serum sample collection

Blood was collected by venipuncture from the saphenous vein on the caudal aspect of the lower leg, placed in serum separating tubes, allowed to clot for at least 30 min, and centrifuged (10 min, 400×*g*). Serum samples were stored at − 70 °C until use. All blood collections were performed on awake animals using a restraint chair and the animals were handled via the pole-and-collar method. Vital signs (pulse, temperature, body weight, and blood pressure) were also measured during each blood collection.

### Serum sample preparation

For both metabolomics and lipidomics, a serum volume of 25 µl was obtained from each sample and transferred to a newly labeled vial. To each aliquot, a volume of 75 µl of extraction solution (35% water, 25% methanol, 40% isopropanol, 0.001% Debrisoquine, 0.005% 4-nitrobenzoic acid) was added. Samples were then vortexed and incubated for 20 min on ice. A volume of 100 µl of chilled acetonitrile was then added to each sample. Samples were vortexed and kept at − 20 °C for 15 min. Next, samples were centrifuged at 15,500×*g* at 4 °C for 20 min. The supernatant from centrifuged samples was transferred to glass vials. To prevent bias, the sample queue was randomized before data acquisition.

### Metabolomics and lipidomics analysis using UPLC-ESI-QTOF-MS

The Acquity UPLC system was used for this study: for metabolomics, an Acquity BEH C18 130 Å, 1.7 µm, 2.1 × 50 mm column was used, and for lipidomics an Acquity UPLC CSH C18 130 Å, 1.7 µm, 2.1 × 100 mm column was used. A 10-min gradient was used to resolve the metabolites with a binary solvent system that included water + 0.1% formic acid (FA), acetonitrile + 0.1% FA, and isopropanol + 0.1% FA. The solvent system used for lipidomics gradient included 50:50 water/acetonitrile + 0.1% FA + 10 mM ammonium formate and 90:10 isopropanol/acetonitrile + 0.1% FA + 10 mM ammonium formate. The ionization source used was electrospray, operating in either positive or negative ionization mode, and the analyzer used was a Xevo G2 quadrupole time-of-flight mass spectrometer. The ESI spray had a positive mode capillary voltage of 3.0 kV, a negative mode a capillary voltage of 2.0 k, and a sampling cone voltage of 30 V in both modes. Desolvation gas flow was set to 1000 l/h and desolvation temperature was set to 500 °C. Cone gas flow was set to 25 l/h and the source temperature was 120 °C. UPLC-QToF-MS acquired centroid mode data with scan time of 0.3 s, interscan time of 0.014 s, and mass range 50–1200 m/z.

Before and after sample runs, a mixture of six standards (acetaminophen: m/z 152.0712 [M+H]^+^/150.0555 [M−H]^−^, sulfaguanidine: m/z 215.0603 [M+H]^+^/213.0446 [M−H]^−^, sulfadimethoxine: m/z 311.0814 [M+H]^+^/309.0658 [M−H]^−^, Val-Tyr-Val: m/z 380.2185 [M+H]^+^/378.2029 [M−H]^−^, terfenadine: m/z 472.3216 [M+H]^+^, and leucine-enkephalin: m/z 556.2771 [M+H]^+^/554.2615 [M−H]^−^) were run to ensure mass accuracy during batch acquisition. Real-time mass correction was applied using a solution of equal parts leucine-enkephalin and aqueous acetonitrile (2.0 ng/ml) at an infusion rate of 10 μl/min utilizing the Waters Lockspray interface. The leucine-enkephalin and aqueous acetonitrile mix was sprayed every 10 s. After every 10 sample injections, a pooled quality control (QC) sample was injected for each group. The pooled QC sample combined aliquots of all samples in the study to represent all metabolites in each matrix and monitor mass accuracy, shifts in retention time, and signal intensities. Pooled QC samples were used to determine reproducibility and data quality—the overlap of QC sample chromatograms (base peak intensity) show minimal shifts in retention time and consistency in peak intensities throughout the acquisition.

### Data processing, statistical analysis, and marker validation

We used the Isotopologue Parameter Optimization (IPO)^27^ R package to initialize and optimize peak picking parameters for our XCMS package^28^ to this specific dataset. Standard XCMS peak pre-processing procedures were applied. Mass spectrometry data, received as mass over charge ratio with retention time, were normalized to internal standards. Additionally, data was normalized based on the QC robust LOESS signal correction^29^. After QC-RLSC, normalized LC–MS data was log transformed and Pareto scaled.

To compare radiation effects on the metabolome and lipidome, an unpaired t test was performed on data separated into groups that were not irradiated (pre-irradiation) and groups that were irradiated. Three unpaired t tests were used to compare Ex-Rad effects on irradiated NHPs: vehicle SD-4 vs pre-irradiation group, Ex-Rad I SD-4 vs vehicle SD-4, and Ex-Rad II SD-4 vs vehicle SD-4. Significantly differentially expressed metabolites with FDR adjusted p value < 0.05 had metabolite identities confirmed using tandem mass spectrometry and NIST 2017 MS/MS spectra database^30^. Fragmentation information of the validated metabolites that were significantly dysregulated for different comparisons are included in Supplementary Table 1. Enrichment analysis was completed via Mummichog v2.0.6 (URL: http://mummichog.org/) to identify metabolic pathways.

### Biomarker discovery for prediction of radiation effect

We used the least absolute shrinkage and selection operator (LASSO)^31^ penalty to perform feature selection, an ROC regularized learning technique. We obtained the regularization path over a grid of values for the tuning parameter lambda through tenfold cross validation, then used the optimal value from cross-validation procedure to fit the model. Therefore, all features with non-zero coefficients were retained in a candidate biomarker panel. This technique is known to reduce over-fitting and variance in classification. The classification performance of the biomarker panel is assessed using the area under the ROC (receiver operating characteristic) curve (AUC).

## Supplementary Information

Supplementary Information 1.
