# Supplementary material for: Analysis of the metabolomic profile in serum of irradiated nonhuman primates treated with Ex-Rad, a radiation countermeasure
Source: Sci Rep. 2021 Jun 1;11:11449. doi: 10.1038/s41598-021-91067-9 (PMC8169671; doi:10.1038/s41598-021-91067-9)
Supplement: Supplementary file 1 — Supplementary Information 1. [file 41598_2021_91067_MOESM1_ESM.pdf]

Supp. Table 1. Table of Tandem MS Validated Metabolites

Precursor m/z	Retention Time	Ionization Mode	Name	Synonyms	Adduct	Level	CID
853.5537	8.29	pos	1-O-octyl-2-O-(N-methylcarbamoyl)-sn-glyceryl-3-phosphorylcholine	Methylcarbamyl PAF C-8	[2M+H]+	A	857.59, 856.58, 855.57, 854.56, 853.55, 797.5, 796.5, 795.5, 794.5, 513.24, 185.07, 184.07, 86.09
162.1130	0.34	pos	N-Acetyl-L-phenylalanine	Acetylphenylalanine	[M+H-CH2O2]+	A	162.11, 120.08
331.2262	2.99	pos	Hydroxyprogesterone	Hydroxyprogesterone	[M+H]+	C	331.23, 313.14
166.0868	0.58	pos	DL-Phenylalanine	Phenylalanine	[M+H]+	A	166.09, 120.08, 103.05
120.0814	0.58	pos	N-(Phenylacetyl)-L-phenylalanine	N-Phenylacetylphenylalanine	[M+H-C3H4O3]+	A	121.08, 120.08, 104.06, 103.06, 93.07
313.2168	3.00	pos	Phe-Phe	Phe-Phe	[M+H]+	C	314.23, 313.18, 120.08,
770.5673	8.63	pos	1-Hexadecyl-2-(8Z,11Z,14Z-eicosatrienoyl)-sn-glycero-3-phosphocholine	PC(O-16:0/20:3)	[M+H]+	A	770.58, 184.07, 104.11, 86.09
121.0846	0.57	pos	Tyramine	Tyramine	[M+H-NH3]+	A	121.08, 120.08, 103.05, 93.07, 77.04
329.2104	2.98	pos	15-Deoxy-.DELTA.12,14-prostaglandin J2 biotinamide	b-15d-PGJ2	[M+2H]2+	B	329.21, 313.13
347.2221	3.02	pos	11-Deoxy-16,16-dimethylprostaglandin E2	11-deoxy-16,16-dimethyl PGE2	[M+H-H2O]+	A	347.22, 329.21
288.2156	3.08	pos	Octanoylcarnitine	Octanoylcarnitine	[M+H]+	A	288.21, 85.02
229.1543	0.35	pos	Leu-Pro	Leu-Pro	[M+H]+	A	229.15, 70.07
311.2008	2.98	pos	Epitestosterone	Epitestosterone	[M+Na]+	B	311.19, 85.03
132.0960	0.40	pos	trans-4-Hydroxy-L-proline	Hydroxyproline	[M+H]+	A	132.08, 86.09
732.5392	8.34	pos	1-Oleoyl-2-myristoyl-sn-glycero-3-phosphocholine	PC(18:1/14:0)	[M+H]+	A	734.57, 733.56, 732.56, 186.08, 185.08, 184.07
204.1108	0.34	pos	Acetyl-DL-carnitine	Acetylcarnitine	[M+H]+	A	205.112, 204.12, 146.06, 145.05, 144.10, 86.02, 85.03
468.3096	4.72	pos	1-Myristoyl-sn-glycero-3-phosphocholine	LysoPC(14:0)	[M+H]+	A	469.29, 468.29, 450.30, 184.07, 104.11, 86.09
794.5454	8.37	pos	1-(1Z-Octadecenyl)-2-(5Z,8Z,11Z,14Z-eicosatetraenoyl)-sn-glycero-3-phosphocholine	PC(P-18:0/20:4)	[M+H]+	A	794.58, 184.07
246.1706	1.76	pos	2-Methylbutyryl-L-carnitine	2-Methylbutyroylcarnitine	[M+H]+	A	246.17, 85.02
746.5580	8.56	pos	1-Hexadecyl-2-(9Z-octadecenoyl)-sn-glycero-3-phosphocholine	PC(O-16:0/18:1)	[M+H]+	A	746.57, 184.07
516.3077	4.87	pos	Oleyloxyethylphosphorylcholine	OOPC	[M+K]+	A	516.31, 457.23, 146.98, 104.11, 86.09, 71.08
494.3250	4.88	pos	1,2-Dipalmitoleoyl-sn-glycero-3-phosphocholine	PC(16:1/16:1)	[M+H]+	A	494.32, 477.31, 476.32, 258.11, 184.07

482.3250	4.97	pos	1-Pentadecanoyl-sn-glycero-3-phosphocholine	LysoPC(15:0)	[M+H]+	A	482.32, 464.31, 299.25, 184.07, 104.11, 86.09
731.5513	8.35	pos	N-(Octadecanoyl)sphing-4-enine-1-phosphocholine	SM(d18:1/18:0)	[M+H]+	A	732.56, 731.55, 184.07, 125.00, 86.09
829.5530	8.57	pos	Lys-Arg-Thr-Leu-Arg-Arg	KRTLRR	[M+H]+	C	831.57, 830.56, 829.55, 795.50, 770.48, 769.48, 674.53, 673.52, 638.46, 517.27,
495.3275	4.88	pos	3-Hydroxybutyrylcarnitine	3-Hydroxybutyrylcarnitine	[2M+H]+	B	495.32, 104.10, 87.10,,86.09, 85.10, 71.08, 60.08, 57.07
400.3419	4.85	pos	Palmitoylcarnitine	Palmitoylcarnitine	[M+H]+	A	400.32, 257.17, 125.08, 85.03
766.5388	8.58	pos	1-O-Hexadecyl-2-O-(5Z,8Z,11Z,14Z,17Z-eicosapentaenoyl)-sn-glyceryl-3-phosphorylcholine	PC(O-16:0/20:5)	[M+H]+	A	769.59, 768.59, 767.57, 766.57, 186.08, 185.08, 184.07
424.3428	4.71	pos	Linoleoylcarnitine	Linoleoylcarnitine	[M+H]+	A	429.30, 239.16, 153.09, 135.09, 125.09, 123.08, 107.09, 97.10, 85.03, 83.09, 69.07
751.5714	8.47	pos	N-Oleoyl-D-erythro-sphingosylphosphorylcholine	SM(d18:1/18:1)	[M+Na]+	A	751.57, 692.50, 568.51, 547.48, 528.52, 184.07, 146.98, 86.09
426.3564	4.94	pos	Oleoyl-L-carnitine	Oleoyl-L-carnitine	[M+H]+	A	426.35, 153.09, 125.09, 107.09, 97.10, 85.03, 83.08, 69.07,
725.5559	8.37	pos	Palmitoyl sphingomyelin	SM(d18:1/16:0)	[M+Na]+	A	725.57, 667.49, 666.48, 543.49, 542.49,
806.5669	8.57	pos	Arachidonoylthiophosphorylcholine	Arachidonoyl Thio-PC	[M+Na]+	A	808.58, 807.57, 806.57, 749.50, 748.49, 747.49, 624.51, 623.50, 601.52, 492.25, 434.26, 433.36, 184.07, 146.98, 86.09, 71.08
118.0869	0.35	pos	4-Amino-2-methylpyrimidine-5-carbonitrile	4-Amino-5-cyano-2-methylpyrimidine	[M+H-NH3]+	A	118.09, 91.05, 77.04,

782.5671	8.69	pos	1,2-Dilinoleoyl-sn-glycero-3-phosphocholine	L-Dilinoleoyllecithin	[M+Na]+	A	785.59, 784.58, 782.57, 724.49, 723.48, 186.08, 185.08, 184.07, 146.98, 125.00, 104.11, 86.09
817.5626	8.67	pos	1-Palmitoyl-2-docosahexaenoyl-sn-glycero-3-phospho-(1'-rac-glycerol)	PG(16:0/22:6)	[M+Na]+	B	820.59, 819.59, 818.59, 817. 59, 648.49, 647.50, 645.48, 623.49
504.3061	4.98	pos	1-Hexadecyl-sn-glycero-3-phosphocholine	PC(O-16:0/0:0)	[M+Na]+	A	504.31, 445.23, 146.98, 104.11,
730.5610	8.37	pos	1,2-Dipalmitoleoyl-sn-glycero-3-phosphocholine	PC(16:1/16:1)	[M+H]+	A	733.56, 732.56, 731.55, 730.56, 186.08, 185.08, 184.07, 125.00, 104.11, 86.09
317.2078	2.17	pos	Val-Ala-Lys	VAK	[M+H]+	B	317.20, 84.08
156.0538	0.31	pos	L-Histidine	Histidine	[M+H]+	A	156.07, 110.07
191.0488	0.32	pos	3,6-Dimethyl-4-hydroxycoumarin	3,6-Dimethyl-4-hydroxycoumarin	[M+H]+	C	191.06, 135.00
192.0407	0.33	pos	2-Nitrobenzaldehyde semicarbazone	2-NP-SCA	[M+H-NH3]+	B	192.06, 135.00, 118.08
135.0035	0.36	pos	1-Phenoxy-2-propanol	1-Phenoxy-2-propanol	[M+H-H2O]+	B	135.00, 91.06
830.5615	8.64	pos	1,2-Diarachidonoyl-sn-glycero-3-phosphocholine	Diarachidonyl lecithin	[M+H]+	A	830.56, 647.50, 526.32, 184.07, 125.00, 104.11, 86.09
764.5231	8.63	pos	2-Docosahexaenoyl-1-palmitoyl-sn-glycero-3-phosphoethanolamine	PE(16:0/22:6)	[M+H]+	A	764.54, 723.50, 436.29, 385.27, 313.27,
256.2147	3.75	pos	2,5-Dimethoxy-4-ethylthioamphetamine	2,5-Dimethoxy-4-ethylthioamphetamine	[M+H]+	C	256.22, 239.16
273.2218	3.75	pos	5-Androstene-3β,17β-diol	Androstenediol	[M+H-H2O]+	A	274.27, 273.21, 256.22, 255.21, 199.15, 161.13, 159.12, 147.12, 145.10
489.2451	3.75	pos	Androstan-3-ol-17-one 3-glucuronide	Etiocholanolone glucuronoside	[M+Na]+	A	489.25, 313.21, 199.02, 163.01
255.2110	3.76	pos	5β-Androsterone	Etiocholanone	[M+H-2H2O]+	A	255.21, 199.15, 173.13, 171.10, 161.13, 159.11, 157.09, 147.12, 145.10, 143.09, 133.11, 131.08, 107.09, 105.07, 97.10, 95.08, 91.05, 83.08, 81.07, 79.05, 69.07

369.3524	8.67	pos	5α-Cholest-7-en-3β-ol	Lathosterol	[M+H-H2O]+	A	369.35., 287.27, 261.25, 259.24, 257.22, 243.21, 233.22, 229.19, 219.21, 217.19, 215.18, 205.19, 203.18, 201.16, 193.19, 189.16, 187.15, 179.18, 177.17, 175.15, 165.17, 163.15, 161.13, 159.12, 149.13, 147.12, 145.10, 137.13, 135.12, 133.10, 123.12, 121.10, 111.12, 109.10, 107.09, 105.07, 95.09, 93.07, 91.05, 83.09, 81.07, 69.07, 67.06
441.2986	8.12	pos	Di(2-nonyl) phthalate	Di(2-nonyl) phthalate	[M+Na]+	B	441.29, 315.16
370.3558	8.67	pos	N-Arachidonylmaleimide	N-Arachidonylmaleimide	[M+H]+	A	370.36, 369.35, 189.16, 175.15, 161.13, 147.12, 135.12, 133.10, 123.12, 119.08, 109.10, 107.09, 105.07, 95.08, 91.05, 83.08, 81.07, 79.05
205.9933	0.41	pos	DL-Indole-3-lactic acid	Indolelactic acid	[M+H]+	A	205.11, 188.07, 118.06
790.5705	8.65	pos	1-Hexadecyl-2-(5Z,8Z,11Z,14Z-eicosatetraenoyl)-sn-glycero-3-phosphocholine	PC(O-16:0/20:4)	[M+Na]+	A	792.58, 791.57, 790.57, 733.50, 732.50, 731.49, 607.50, 585.52, 146.98, 86.09, 71.08
786.5061	8.63	pos	1,2-dioleoyl-sn-glycero-3-phosphatidylcholine	PC(18:1/18:1)	[M+H]+	A	786.56, 522.36, 185.07, 184.07
212.0949	2.73	pos	Kynurenic acid	Kynurenic acid	[M+Na]+	A	212.02, 211.09, 168.00
757.5559	8.44	pos	Thioetheramidephosphatidylcholine	Thioetheramidephosphatidylcholine	[M+Na]+	A	757.55, 698.48, 574.49, 271.07, 184.07, 146.98, 104.11, 86.09
756.5526	8.44	pos	1,2-Dihexadecanoyl-sn-glycero-3-phosphocholine	Colfosceril palmitate	[M+Na]+	A	756.55, 698.48, 697.47, 573.48, 184.07, 125.00, 86.09

257.1752	3.56	pos	5α-Androstane-3α,17β-diol	Androstanediol	[M+H-2H2O]+	A	257.18, 175.15, 161.13, 147.12, 135.11, 121.10, 107.08, 97.10, 83.08, 79.05, 69.07
132.9935	0.36	pos	L-Methionine	Methionine	[M+H-NH3]+	A	132.09, 86.09
520.5097	8.35	pos	N-Palmitoyl-D-sphingosine	D-erythro-C16-Ceramide	[M+H-H2O]+	A	520.51, 502.49, 264.27
447.3356	7.82	pos	Lyso-sphingomyelin	LysoSM(d18:1)	[M+H]+	B	447.33, 184.07
729.5890	8.47	pos	N-Oleoyl-D-erythro-sphingosylphosphorylcholine	SM(d18:1/18:1)	[M+H]+	A	729.59, 184.07, 125.00, 86.09
188.0711	0.93	pos	DL-Indole-3-lactic acid	Indolelactic acid	[M+H-H2O]+	A	188.07, 170.06, 146.06, 143.07, 142.06, 118.07, 117.06, 115.06, 91.05,
229.1440	3.05	pos	Zebularine	4-Deoxyuridine	[M+H]+	C	229.14, 97.06
788.5214	8.62	pos	1-Octadecanoyl-2-octadecenoyl-sn-glycero-3-phosphocholine	PC(18:0/18:1)	[M+H]+	A	788.56, 787.56, 184.07, 125.00, 86.09
159.0915	0.93	pos	L-Propionylcarnitine	Propionylcarnitine	[M+H-C3H9N]+	B	159.12, 158.12, 90.98, 85.02
146.0519	0.93	pos	L-Kynurenine	L-Kynurenine	[M+H]+	A	146.09, 118.07
184.0740	8.37	pos	Phosphocholine	Phosphocholine	[M+H]+	A	184.07, 125.00, 98.98, 86.09, 71.07
489.2535	4.52	pos	Androstan-3-ol-17-one 3-glucuronide	Etiocholanolone Glucuronide	[M+Na]+	A	489.25, 313.21, 199.02
483.2290	4.53	pos	1-Stearoyl-2-hydroxy-sn-glycero-3-phosphate	LysoPA(18:0)	[M-H+2Na]+	A	483.26, 184.07, 125.02
874.5602	8.32	neg	1,2-Diarachidonoyl-sn-glycero-3-phosphocholine	Diarachidonyl lecithin	[M+HCO2]-	B	876.58, 816.55
184.0967	0.55	pos	1,2-Dipalmitoyl-sn-glycero-O-ethyl-3-phosphatidylcholine cation	1,2-Dipalmitoyl-sn-glycero-O-ethyl-3-PC cation	[Cat-C37H70O4]+	A	184.07, 124.99, 86.096
162.1130	0.52	pos	L-Carnitine	Carnitine	[M+H]+	A	162.11, 103.04, 102.09, 85.03
468.3093	0.91	pos	1-Myristoyl-sn-glycero-3-phosphocholine	LysoPC(14:0)	[M+H]+	A	468.30, 184.07, 125.01, 104.11, 86.09
784.5746	4.37	pos	Arachidonoylthiophosphorylcholine	Arachidonoyl Thio-PC	[M+H]+	B	784.57, 184.07, 86.09
454.2936	1.25	pos	1-Palmitoyl-2-hydroxy-sn-glycero-3-phosphoethanolamine	LysoPE(16:0)	[M+H]+	A	454.28, 436.28, 313.27
830.5681	4.20	pos	1,2-Diarachidonoyl-sn-glycero-3-phosphocholine	Diarachidonyl lecithin	[M+H]+	A	830.57, 184.07, 86.09
482.3249	1.02	pos	1-Pentadecanoyl-sn-glycero-3-phosphocholine	LysoPC(15:0)	[M+H]+	A	482.32, 464.32, 184.07, 104.11
494.3247	0.96	pos	1,2-Dipalmitoleoyl-sn-glycero-3-phosphocholine	PC(16:1/16:1)	[M+H]+	A	494.32, 476.31, 258.11, 184.07
132.0735	0.52	pos	N-Acetyl-L-alanine	Acetylalanine	[M+H]+	B	132.07, 90.05, 86.09
120.0814	0.58	pos	(2-Mercaptoethyl)guanidine	2-Mercaptoethylguanidine	[M+H]+	A	120.08, 119.08, 103.05
548.3719	1.37	pos	1-O-Octadecyl-sn-glyceryl-3-phosphorylcholine	LysoPC(O-18:0)	[M+K]+	A	548.34, 489.29, 104.11

510.3560	1.40	pos	1-Heptadecanoyl-sn-glycero-3-phosphocholine	LysoPC(17:0)	[M+H]+	A	510. 36, 492.34, 184.07, 125.09, 104.11, 86.09
544.3378	1.27	pos	1-Oleoyl-sn-glycero-3-phosphocholine	LysoPC(18:1)	[M+Na]+	A	544.34, 488.28, 485.26, 484.25, 339.29, 146.98, 105.11, 86.09
756.5519	4.92	pos	1,2-Dihexadecanoyl-sn-glycero-3-phosphocholine	PC(16:0/16:0)	[M+Na]+	A	756.55, 698.48, 697.47, 574.49, 573.49, 184.07, 146.98, 86.09
522.3560	1.27	pos	1-Oleoyl-sn-glycero-3-phosphocholine	LysoPC(18:1)	[M+H]+	A	523.36, 522.35, 505.35, 504.35, 340.29, 339.29, 258.11, 184.07, 125.00, 104.11, 86.09
731.5408	4.17	pos	N-(Octadecanoyl)sphing-4-enine-1-phosphocholine	SM(d18:1/18:0)	[M+H]+	A	731.50, 184.07, 86.09,
742.5689	4.87	pos	1,2-Dipalmitoyl-sn-glycero-3-phosphoethanolamine-N,N-dimethyl	DMPE(16:0/16:0)	[M+Na]+	B	742.57, 601.51
118.0873	0.52	pos	L-Valine	Valine	[M+H]+	A	118.09, 72.08
140.0692	0.54	pos	Picolinic acid N-oxide	Picolinic acid N-oxide	[M+H]+	B	140.07, 96.08
787.5936	4.76	pos	N-Docosanoyl-4-sphingenyl-1-O-phosphorylcholine	SM(d18:1/22:0)	[M+H]+	A	786.60, 786.60, 184.07, 86.09
735.5718	4.94	pos	Thioetheramidephosphatidylcholine	Thioetheramide-PC	[M+H]+	A	735.57, 184.07
548.3603	1.68	pos	1-O-Octadecyl-sn-glyceryl-3-phosphorylcholine	LysoPC(O-18:0)	[M+K]+	A	548.34, 489.29, 104.11
431.3888	4.85	pos	(+)-α-Tocopherol	Vitamin E	[M+H]+	A	431.38, 430.38, 166.09, 165.09, 164.08
734.5688	4.94	pos	1,2-Dipalmitoyl-sn-glycero-O-ethyl-3-phosphatidylcholine cation	1,2-Dipalmitoyl-sn-glycero-O-ethyl-3-PC cation	[Cat-C2H4]+	A	734.57,184.07
562.3288	1.68	pos	1-O-Hexadecyl-2-deoxy-2-thio-S-acetyl-sn-glyceryl-3-phosphorylcholine	2-Thio PAF	[M+Na]+	A	562.32, 146.98, 104.11, 86.09
524.3717	1.68	pos	1-Stearoyl-2-hydroxy-sn-glycero-3-phosphocholine	LysoPC(18:0)	[M+H]+	A	524.37, 506.36, 447.29, 341.31, 258.11, 184.07, 125.00, 104.11, 86.09
786.5904	4.76	pos	1-Stearoyl-2-linoleoyl-sn-glycero-3-phosphocholine	PC(18:0/18:2)	[M+H]+	A	786.60, 184.07, 125.00, 104.11, 86.09
546.3560	1.14	pos	1-Stearoyl-2-hydroxy-sn-glycero-3-phosphocholine	LysoPC(18:0)	[M+Na]+	A	546.35, 487.28, 443.26, 341.31, 146.98, 104.11, 97.10, 95.09, 86.09, 71.08
156.0490	0.56	pos	L-Histidine	Histidine	[M+H]+	A	156.06, 110.07
546.3535	1.68	pos	1-Stearoyl-2-hydroxy-sn-glycero-3-phosphocholine	LysoPC(18:0)	[M+Na]+	A	546.35, 487.28, 443.26, 341.31, 146.98, 104.11, 86.09
506.3595	1.68	pos	1-Myristoyl-sn-glycero-3-phosphocholine	LysoPC(14:0)	[M+K]+	A	506.37, 184.07, 104.11, 86.09

764.5216	4.62	pos	2-Docosahexaenoyl-1-palmitoyl-sn-glycero-3-phosphoethanolamine	PE(16:0/22:6)	[M+H]+	B	764.54, 624.51, 623.51
520.3404	1.03	pos	1,2-Dilinoleoyl-sn-glycero-3-phosphocholine	L-Dilinoleoyllecithin	[M+H]+	A	520.34, 443.25, 337.27, 258.11, 184.07, 166.06
768.5526	4.37	pos	1-Hexadecyl-2-(5Z,8Z,11Z,14Z-eicosatetraenoyl)-sn-glycero-3-phosphocholine	PC(O-16:0/20:4)	[M+H]+	A	768.54, 186.08, 185.08, 184.07, 146.98, 86.09
568.3399	0.97	pos	1-Docosahexaenoyl-2-stearoyl-sn-glycero-3-phosphocholine	1-Docosahexaenoyl-2-stearoyl-sn-glycero-3-phosphocholine	[M+H]+	A	568.34, 551.32, 550.33, 184.07
790.5709	4.89	pos	1-Hexadecyl-2-(5Z,8Z,11Z,14Z-eicosatetraenoyl)-sn-glycero-3-phosphocholine	PC(O-16:0/20:4)	[M+Na]+	A	791.58, 790.57, 732.50, 731.50, 608.51, 607.51, 585.52
478.2944	1.07	pos	1-Palmitoyl-sn-glycero-3-phosphocholine	LysoPC(16:0)	[M+H-H2O]+	A	478.31, 419.25, 184.07, 181.03, 163.03, 125.00, 86.09,
786.5999	5.11	pos	1,2-dioleoyl-sn-glycero-3-phosphatidylcholine	DOPC	[M+H]+	A	787.60, 786.60, 785.59, 185.08, 184.07, 125.00, 104.11,
787.6028	5.11	pos	N-Docosanoyl-4-sphingenyl-1-O-phosphorylcholine	SM(d18:1/22:0)	[M+H]+	A	787.60, 786.60, 184.07, 125.00, 86.09
590.3227	0.97	pos	1-Octadecyl-2-acetyl-sn-glycero-3-phosphocholine	PC(O-18:0/2:0)	[M+K]+	A	590.32, 532.25, 531.25, 407.26, 86.09
810.5996	5.05	pos	1-Stearoyl-2-linoleoyl-sn-glycero-3-phospho-L-serine	PS(18:0/18:2)	[M+Na]+	A	812.60, 811.60, 810.59, 626.52, 604.54, 603.54, 625.51, 604.54, 603.54
522.3463	1.02	pos	1-Oleoyl-sn-glycero-3-phosphocholine	LysoPC(18:1)	[M+H]+	A	523.36, 522.35, 505.35, 504.35, 340.29, 339.29, 445.27, 258.11, 185.08, 184.07, 125.00, 105.11, 104.11, 86.09
482.3606	1.38	pos	1-Pentadecanoyl-sn-glycero-3-phosphocholine	LysoPC(15:0)	[M+H]+	A	482.32, 464.31, 184.07, 104.11, 86.09
762.5895	4.99	pos	1-Palmitoyl-2-stearoyl-sn-glycero-3-phosphocholine	PC(16:0/18:0)	[M+H]+	A	763.59, 762.59, 761.59, 185.08, 184.07,
439.2300	0.66	pos	Oleoyl 3-carbacyclic phosphatidic acid	Oleoyl 3-carbacyclic phosphatidic acid	[M+Na]+	A	440.25, 439.24, 438.20, 211.09
508.3754	1.47	pos	1-(1Z-Octadecenyl)-sn-glycero-3-phosphocholine	LysoPC(P-18:0)	[M+H]+	A	508.38, 184.07, 104.11, 86.09
716.5243	4.79	pos	2-Linoleoyl-1-palmitoyl-sn-glycero-3-phosphoethanolamine	PE(16:0/18:2)	[M+H]+	A	716.52, 575.51

524.2773	1.01	neg	1-(1Z-Hexadecenyl)-sn-glycero-3-phosphocholine	LysoPC(P-16:0)	[M+HCO2]-	B	524.29, 464.31
862.5529	4.53	neg	1-(1Z-Octadecenyl)-2-(4Z,7Z,10Z,13Z,16Z,19Z-docosahexaenoyl)-sn-glycero-3-phosphocholine	PC(P-18:0/22:6)	[M+HCO2]-	B	862.55, 802.57
512.2988	0.92	neg	1-Myristoyl-sn-glycero-3-phosphocholine	LysoPC(14:0)	[M+HCO2]-	A	512.29, 452.28, 227.20
788.5236	4.74	neg	2-Oleoyl-1-stearoyl-sn-glycero-3-phosphoserine	PS(18:0/18:1)	[M-H]-	B	788.53, 283.26
816.5754	4.95	neg	1-(1Z-Octadecenyl)-2-(9Z-octadecenoyl)-sn-glycero-3-phosphocholine	PC(P-18:0/18:1)	[M+HCO2]-	B	816.57, 756.56
778.5597	4.99	neg	1-Myristoyl-2-stearoyl-sn-glycero-3-phosphocholine	PC(14:0/18:0)	[M+HCO2]-	B	778.57, 718.54
850.5605	4.56	neg	1-Palmitoyl-2-docosahexaenoyl-sn-glycero-3-phosphocholine	PC(16:0/22:6)	[M+HCO2]-	A	850.56, 790.53, 327.23, 283.26, 255.23
826.5606	4.66	neg	1-Palmitoyl-2-arachidonoyl-sn-glycero-3-phosphocholine	PC(16:0/20:4)	[M+HCO2]-	A	826.56, 766.54, 480.31, 303.23, 259.24, 255.23
465.2483	0.63	neg	Androstan-3-ol-17-one 3-glucuronide	Etiocholanolone Glucuronide	[M-H]-	B	465.25, 113.01, 85.03
201.0380	0.58	neg	Ile-Ala	Ile-Ala	[M-H]-	A	201.80, 199.80, 166.83, 164.83, 162.84,
747.5158	4.89	neg	Palmitoyl sphingomyelin	SM(d18:1/16:0)	[M+HCO2]-	B	747.55, 687.54, 168.04
747.5657	4.47	neg	Palmitoyl sphingomyelin	SM(d18:1/16:0)	[M+HCO2]-	A	747.57, 687.54, 168.04,
738.5075	4.80	neg	2-Arachidonoyl-1-palmitoyl-sn-glycero-3-phosphoethanolamine	PE(16:0/20:4)	[M-H]-	A	741.52, 740.52, 738.50, 303.23, 255.23
838.5958	4.97	neg	1-(1Z-Octadecenyl)-2-(5Z,8Z,11Z,14Z-eicosatetraenoyl)-sn-glycero-3-phosphocholine	PC(P-18:0/20:4)	[M+HCO2]-	B	838.59, 778.58
186.0432	0.58	neg	2-Hydroxy-6-methylquinoline-3-carbaldehyde	6-methyl-2-oxo-1H-quinoline-3-carbaldehyde	[M-H]-	B	186.04, 115.02, 114.02,
862.5955	4.87	neg	1-(1Z-Octadecenyl)-2-(4Z,7Z,10Z,13Z,16Z,19Z-docosahexaenoyl)-sn-glycero-3-phosphocholine	PC(P-18:0/22:6)	[M+HCO2]-	B	862.59, 802.58
816.5553	4.47	neg	1-(1Z-Octadecenyl)-2-(9Z-octadecenoyl)-sn-glycero-3-phosphocholine	PC(P-18:0/18:1)	[M+HCO2]-	B	816.57, 756.55
885.5502	4.49	neg	1-Stearoyl-2-arachidonoyl-sn-glycero-3-phospho-(1'-myo-inositol)	PI(18:0/20:4)	[M-H]-	A	885.55, 581.31, 419.25, 303.23, 283.26, 241.01
878.5916	5.01	neg	1-Stearoyl-2-docosahexaenoyl-sn-glycero-3-phosphocholine	PC(18:0/22:6)	[M+HCO2]-	A	878.59, 818.57, 508.34, 327.23, 283.26
609.3207	1.21	neg	1-Myristoyl-sn-glycero-3-phosphocholine	LysoPC(14:0)	[M+HCO2]-	B	512.29, 452.28, 227.20

Supp. Table 2. Test Statistics for tandem MS validated metabolites radiation effect and Ex-Rad protection effect																			
				Vehicle SD 1 vs. Pre-irradiation				Vehicle SD 4 vs. Pre-irradiation				Ex-Rad1 SD 4 vs. Vehicle SD 4				Ex-Rad2 SD 4 vs. Vehicle SD 4			
Name	*Seq*	*m/z*	*RT*	*p-value*	*FDR*	*FC*	*Log2FC*	*p-value*	*FDR*	*FC*	*Log2FC*	*p-value*	*FDR*	*FC*	*Log2FC*	*p-value*	*FDR*	*FC*	*Log2FC*
1,2-Dipalmitoyl-sn-glycero-O-ethyl-3-PC	73	734.5688	4.94	3.05E-14	1.16E-12	3.20 ↑	1.68	2.68E-07	1.28E-05	2.45 ↑	1.29	1.04E-01	5.06E-01	1.51 ↑	0.60	4.36E-01	6.71E-01	0.87 ↓	-0.20
11-deoxy-16,16-	10	347.2221	3.02	1.26E-03	5.52E-03	1.44 ↑	0.52	6.84E-03	4.44E-02	1.87 ↑	0.90	1.45E-02	1.52E-01	0.48 ↓	-1.06	1.09E-02	1.06E-01	0.45 ↓	-1.16
1-Docosahexaenoyl-2-stearoyl-sn-glycero-3-	107	568.3399	0.97	3.55E-05	2.36E-04	1.57 ↑	0.65	1.78E-04	2.41E-03	1.87 ↑	0.90	4.26E-01	8.99E-01	1.21 ↑	0.28	3.83E-01	6.30E-01	0.82 ↓	-0.29
1-Phenoxy-2-propanol	43	135.0035	0.36	1.41E-03	6.07E-03	1.41 ↑	0.50	9.82E-03	5.74E-02	1.86 ↑	0.89	2.22E-02	2.02E-01	0.49 ↓	-1.04	1.68E-02	1.37E-01	0.45 ↓	-1.14
2-	19	246.1706	1.76	1.40E-03	6.05E-03	1.41 ↑	0.50	6.50E-03	4.28E-02	1.82 ↑	0.86	1.57E-02	1.59E-01	0.50 ↓	-1.01	1.31E-02	1.17E-01	0.47 ↓	-1.08
2-Thio PAF	97	562.3288	1.68	3.54E-08	3.77E-07	1.65 ↑	0.72	9.40E-04	9.14E-03	1.63 ↑	0.71	2.23E-01	7.55E-01	1.28 ↑	0.35	7.76E-01	8.91E-01	0.90 ↓	-0.15
3-	26	495.3275	4.88	7.84E-03	2.38E-02	1.24 ↑	0.31	1.43E-05	1.44E-04	1.53 ↑	0.62	6.71E-02	5.55E-01	0.80 ↓	-0.33	8.47E-03	2.48E-01	0.70 ↓	-0.52
4-Deoxyuridine	65	229.144	3.05	4.25E-18	3.53E-16	1.89 ↑	0.92	8.78E-07	4.00E-05	1.51 ↑	0.60	8.74E-01	9.28E-01	1.03 ↑	0.04	5.02E-01	7.65E-01	0.96 ↓	-0.06
Acetylalanine	81	132.0735	0.52	1.19E-11	2.27E-10	1.91 ↑	0.93	2.10E-03	1.81E-02	1.50 ↑	0.58	7.20E-02	4.12E-01	1.48 ↑	0.57	5.00E-01	7.20E-01	1.06 ↑	0.09
Acetylcarnitine	16	204.1108	0.34	1.06E-06	8.99E-06	1.59 ↑	0.67	2.25E-02	1.06E-01	1.49 ↑	0.57	2.73E-01	7.98E-01	1.20 ↑	0.27	2.25E-01	4.82E-01	0.76 ↓	-0.39
Acetylphenylalanine	2	162.113	0.34	5.93E-03	1.85E-02	1.27 ↑	0.34	8.61E-03	3.48E-02	1.42 ↑	0.51	9.89E-01	9.96E-01	0.94 ↓	-0.08	2.85E-01	8.84E-01	0.80 ↓	-0.33
Androstenediol	47	273.2218	3.75	1.16E-07	9.82E-07	1.31 ↑	0.39	5.22E-06	1.08E-04	1.41 ↑	0.50	5.08E-01	7.86E-01	1.07 ↑	0.10	4.72E-01	7.62E-01	0.93 ↓	-0.10
Arachidonoyl Thio-PC	33	806.5669	8.57	4.49E-15	2.19E-13	1.91 ↑	0.93	6.52E-03	4.28E-02	1.39 ↑	0.48	1.14E-01	5.24E-01	1.36 ↑	0.45	4.39E-01	6.75E-01	0.87 ↓	-0.20
D-erythro-C16-	61	520.5097	8.35	2.45E-07	1.94E-06	1.22 ↑	0.28	2.13E-05	2.92E-04	1.33 ↑	0.41	7.60E-01	8.83E-01	1.02 ↑	0.03	7.95E-01	9.17E-01	0.95 ↓	-0.07
Diarachidonyl lecithin	44	874.5602	8.32	1.39E-12	3.28E-11	1.44 ↑	0.52	3.24E-04	3.77E-03	1.32 ↑	0.40	7.46E-01	9.70E-01	1.12 ↑	0.17	4.04E-01	6.44E-01	0.91 ↓	-0.13
Dilinoleoyllecithin	35	782.5671	8.69	4.66E-01	7.18E-01	1.05 ↑	0.07	4.95E-02	1.70E-01	1.15 ↑	0.20	5.47E-04	1.41E-02	0.75 ↓	-0.42	1.47E-01	3.89E-01	0.90 ↓	-0.15
DMPE(16:0/16:0)	89	742.5689	4.87	1.98E-26	6.97E-24	2.48 ↑	1.31	7.33E-02	1.91E-01	1.31 ↑	0.39	8.56E-02	5.55E-01	1.25 ↑	0.32	2.86E-01	8.84E-01	1.12 ↑	0.17
DOPC	110	786.5999	5.11	3.41E-24	7.65E-22	2.44 ↑	1.29	9.84E-03	5.74E-02	1.31 ↑	0.39	4.20E-01	8.97E-01	1.11 ↑	0.15	1.98E-01	4.51E-01	1.16 ↑	0.22
Epitestosterone	13	311.2008	2.98	8.32E-09	7.90E-08	1.64 ↑	0.72	8.49E-02	2.15E-01	1.27 ↑	0.34	2.28E-01	5.71E-01	1.32 ↑	0.40	3.48E-01	8.84E-01	1.14 ↑	0.19
Etiocholanolone	48	489.2451	3.75	2.08E-14	8.18E-13	1.91 ↑	0.93	4.60E-02	1.62E-01	1.25 ↑	0.33	2.47E-01	7.74E-01	1.27 ↑	0.34	3.15E-01	5.80E-01	0.86 ↓	-0.22
Etiocholanone	49	255.211	3.76	9.98E-08	9.74E-07	1.20 ↑	0.27	2.90E-05	5.66E-04	1.25 ↑	0.32	3.79E-01	8.78E-01	1.08 ↑	0.11	9.02E-01	9.52E-01	0.99 ↓	-0.01
Hydroxyprogesterone	3	331.2262	2.99	1.57E-02	4.08E-02	1.07 ↑	0.10	2.89E-01	4.12E-01	1.19 ↑	0.26	4.73E-01	7.75E-01	0.84 ↓	-0.26	2.51E-01	7.54E-01	0.79 ↓	-0.33
Hydroxyproline	14	132.096	0.4	1.47E-02	4.22E-02	1.26 ↑	0.33	3.44E-01	5.47E-01	1.19 ↑	0.25	1.35E-01	5.55E-01	1.20 ↑	0.27	5.04E-01	9.02E-01	1.03 ↑	0.04
Indolelactic acid	53	188.0711	0.93	9.95E-08	8.56E-07	1.32 ↑	0.40	1.71E-01	3.05E-01	1.17 ↑	0.23	3.71E-01	7.55E-01	0.86 ↓	-0.22	4.35E-01	7.61E-01	0.87 ↓	-0.21
KRTLRR	25	829.553	8.57	2.53E-07	2.34E-06	1.18 ↑	0.24	1.69E-03	1.50E-02	1.17 ↑	0.22	2.39E-01	7.73E-01	1.10 ↑	0.14	3.29E-01	5.90E-01	1.06 ↑	0.08
Kynurenic acid	56	212.0949	2.73	8.89E-17	7.13E-15	1.34 ↑	0.43	5.65E-04	5.90E-03	1.16 ↑	0.21	1.88E-01	6.95E-01	0.93 ↓	-0.11	4.19E-03	5.47E-02	0.86 ↓	-0.21
Lathosterol	50	369.3524	8.67	1.20E-15	6.94E-14	1.38 ↑	0.47	1.03E-03	9.84E-03	1.16 ↑	0.21	3.68E-01	8.75E-01	0.96 ↓	-0.06	3.35E-03	4.67E-02	0.86 ↓	-0.21
L-Carnitine	74	162.113	0.52	1.40E-13	4.07E-12	1.87 ↑	0.91	3.63E-02	1.41E-01	1.34 ↑	0.42	9.15E-02	4.70E-01	1.41 ↑	0.49	7.00E-01	8.59E-01	0.89 ↓	-0.16
L-Histidine	40	156.0538	0.31	3.75E-02	8.42E-02	1.09 ↑	0.13	8.33E-04	4.93E-03	1.25 ↑	0.32	8.45E-01	9.17E-01	1.02 ↑	0.03	2.39E-01	7.54E-01	0.91 ↓	-0.13
Linoleoylcarnitine	29	424.3428	4.71	3.02E-10	4.47E-09	1.16 ↑	0.21	1.05E-04	1.61E-03	1.14 ↑	0.19	2.89E-01	8.06E-01	1.05 ↑	0.07	8.69E-01	9.37E-01	1.01 ↑	0.01
L-Kynurenine	68	146.0519	0.93	3.53E-01	6.11E-01	1.07 ↑	0.09	4.99E-02	1.70E-01	1.14 ↑	0.19	8.28E-04	1.81E-02	0.77 ↓	-0.37	1.43E-01	3.86E-01	0.91 ↓	-0.13
L-Methionine	60	132.9935	0.36	3.33E-04	1.40E-03	0.86 ↓	-0.22	7.04E-01	8.01E-01	1.04 ↑	0.05	2.90E-01	6.34E-01	0.91 ↓	-0.14	2.02E-03	9.16E-02	0.72 ↓	-0.47
L-Phenylalanine	4	166.0868	0.58	4.80E-02	1.34E-01	1.14 ↑	0.18	2.44E-04	3.07E-03	0.79 ↓	-0.34	1.66E-01	6.49E-01	1.18 ↑	0.24	1.79E-04	4.96E-03	1.43 ↑	0.51
LysoPA(18:0)	71	483.229	4.53	4.49E-01	7.07E-01	1.06 ↑	0.09	5.42E-02	1.76E-01	1.13 ↑	0.17	2.88E-03	4.51E-02	0.81 ↓	-0.31	1.46E-01	3.87E-01	0.93 ↓	-0.11
LysoPC(14:0)	17	468.3093	0.91	4.04E-03	1.35E-02	0.88 ↓	-0.18	1.37E-01	3.03E-01	1.12 ↑	0.16	7.91E-02	5.55E-01	0.84 ↓	-0.26	4.01E-04	3.52E-02	0.68 ↓	-0.56
LysoPC(14:0)	17	506.3595	1.68	8.30E-01	9.01E-01	1.02 ↑	0.04	1.13E-01	2.66E-01	1.12 ↑	0.16	1.18E-03	8.32E-02	0.73 ↓	-0.46	1.47E-05	5.17E-03	0.61 ↓	-0.72
LysoPC(15:0)	23	482.3606	1.38	3.47E-01	5.54E-01	0.95 ↓	-0.08	1.89E-01	3.75E-01	1.11 ↑	0.16	1.05E-02	3.49E-01	0.75 ↓	-0.42	3.21E-04	3.23E-02	0.65 ↓	-0.62
LysoPC(16:0)	109	478.2944	1.07	6.06E-01	7.56E-01	1.00 ↓	0.00	8.77E-01	9.12E-01	1.10 ↑	0.14	1.65E-04	1.94E-02	1.98 ↑	0.99	7.20E-01	9.25E-01	0.88 ↓	-0.19
LysoPC(17:0)	84	510.356	1.4	1.67E-02	4.25E-02	0.84 ↓	-0.26	7.31E-01	8.09E-01	1.09 ↑	0.13	6.04E-01	8.22E-01	0.88 ↓	-0.18	9.46E-01	9.81E-01	0.92 ↓	-0.12
LysoPC(18:0)	98	524.3717	1.68	3.72E-01	6.31E-01	1.09 ↑	0.13	9.99E-01	9.99E-01	1.08 ↑	0.11	1.16E-09	2.07E-06	3.74 ↑	1.90	4.16E-08	3.11E-05	2.73 ↑	1.45
LysoPC(18:1)	85	522.356	1.27	1.05E-02	3.75E-02	0.88 ↓	-0.19	7.43E-01	8.81E-01	1.07 ↑	0.10	5.47E-02	3.46E-01	1.27 ↑	0.34	4.47E-01	6.80E-01	0.85 ↓	-0.24
LysoPC(18:1) isomer	85	522.3463	1.02	1.21E-05	6.47E-05	0.79 ↓	-0.35	5.73E-01	7.24E-01	1.07 ↑	0.10	2.03E-01	5.55E-01	1.13 ↑	0.17	7.46E-01	9.28E-01	0.94 ↓	-0.09
LysoPC(O-18:0)	83	548.3603	1.68	3.95E-18	4.22E-16	1.92 ↑	0.94	7.88E-01	9.15E-01	1.06 ↑	0.08	8.06E-01	9.72E-01	1.02 ↑	0.03	1.83E-02	1.45E-01	0.73 ↓	-0.46
LysoPC(P-16:0)	120	524.2773	1.01	6.45E-01	8.61E-01	1.06 ↑	0.08	8.43E-01	9.38E-01	1.05 ↑	0.08	1.38E-04	5.24E-03	2.35 ↑	1.23	8.15E-01	9.09E-01	1.01 ↑	0.01

LysoPC(P-18:0)	118	508.3754	1.47	1.26E-02	4.40E-02	1.05 ↑	0.08	8.96E-02	2.42E-01	1.05 ↑	0.07	5.64E-01	9.58E-01	1.02 ↑	0.03	8.50E-01	9.29E-01	1.01 ↑	0.01
LysoPE(16:0)	77	454.2936	1.25	1.10E-11	2.15E-10	1.18 ↑	0.23	1.13E-01	2.80E-01	1.05 ↑	0.07	3.50E-02	2.63E-01	1.09 ↑	0.12	3.52E-09	7.90E-06	1.73 ↑	0.79
LysoSM(d18:1)	62	447.3356	7.82	1.32E-03	4.95E-03	0.88 ↓	-0.18	5.86E-01	7.28E-01	1.05 ↑	0.07	3.51E-01	6.70E-01	0.92 ↓	-0.12	3.99E-03	1.56E-01	0.75 ↓	-0.41
Methylcarbamyl PAF C-8	1	853.5537	8.29	2.73E-03	1.11E-02	1.08 ↑	0.12	7.06E-01	8.68E-01	1.01 ↑	0.02	1.36E-01	5.89E-01	1.06 ↑	0.09	3.66E-01	6.20E-01	0.96 ↓	-0.06
N-Arachidonylmaleimide	52	370.3558	8.67	1.35E-03	4.99E-03	0.83 ↓	-0.26	3.59E-01	5.52E-01	1.01 ↑	0.02	9.51E-01	9.84E-01	0.89 ↓	-0.17	2.89E-01	8.84E-01	0.74 ↓	-0.44
Octanoylcarnitine	11	288.2156	3.08	1.21E-10	1.89E-09	1.73 ↑	0.79	7.52E-01	8.88E-01	1.01 ↑	0.01	4.29E-01	8.99E-01	1.11 ↑	0.15	9.37E-01	9.69E-01	0.98 ↓	-0.03
Oleoyl 3-carbacyclic phosphatidic acid	117	439.23	0.66	1.09E-26	2.32E-23	0.50 ↓	-0.99	6.64E-01	8.85E-01	0.99 ↓	-0.02	6.47E-01	9.59E-01	0.96 ↓	-0.05	3.73E-02	2.65E-01	0.79 ↓	-0.34
Oleoyl-L-carnitine	31	426.3564	4.94	9.39E-05	5.48E-04	0.79 ↓	-0.35	3.47E-01	5.86E-01	0.95 ↓	-0.07	4.94E-02	3.24E-01	0.74 ↓	-0.43	2.35E-01	4.90E-01	0.83 ↓	-0.27
OOPC	21	516.3077	4.87	1.07E-05	5.76E-05	1.26 ↑	0.33	1.40E-01	3.07E-01	0.95 ↓	-0.08	1.57E-01	5.55E-01	1.14 ↑	0.18	3.05E-01	8.84E-01	1.09 ↑	0.12
Palmitoylcarnitine	27	400.3419	4.85	1.60E-05	8.22E-05	1.24 ↑	0.31	1.04E-01	2.54E-01	0.94 ↓	-0.09	1.33E-01	5.55E-01	1.15 ↑	0.20	2.77E-01	8.84E-01	1.10 ↑	0.13
PC(14:0/18:0)	125	778.5597	4.99	1.35E-05	9.71E-05	0.94 ↓	-0.09	4.93E-04	5.25E-03	0.93 ↓	-0.10	7.78E-01	9.72E-01	1.01 ↑	0.01	5.68E-02	2.71E-01	1.04 ↑	0.06
PC(16:0/16:0)	86	756.5519	4.92	3.47E-07	3.15E-06	0.84 ↓	-0.25	1.18E-01	2.87E-01	0.93 ↓	-0.10	9.15E-02	4.70E-01	0.90 ↓	-0.16	7.01E-06	5.25E-04	0.71 ↓	-0.49
PC(16:0/18:0)	116	762.5895	4.99	1.69E-05	1.19E-04	0.93 ↓	-0.10	7.89E-04	7.77E-03	0.93 ↓	-0.10	4.82E-01	9.28E-01	1.02 ↑	0.03	2.39E-02	1.74E-01	1.05 ↑	0.07
PC(16:0/20:4)	127	826.5606	4.66	5.28E-07	4.73E-06	0.80 ↓	-0.32	2.73E-01	5.03E-01	0.93 ↓	-0.11	3.42E-01	8.48E-01	0.93 ↓	-0.11	5.04E-01	7.22E-01	1.06 ↑	0.08
PC(16:0/22:6)	126	850.5605	4.56	4.63E-03	1.78E-02	0.89 ↓	-0.17	2.34E-01	4.53E-01	0.92 ↓	-0.12	1.50E-01	6.21E-01	0.91 ↓	-0.13	1.19E-01	3.59E-01	1.10 ↑	0.13
PC(16:1/16:1)	22	730.561	8.37	1.42E-08	1.60E-07	0.63 ↓	-0.67	4.62E-01	6.94E-01	0.92 ↓	-0.12	8.49E-03	1.08E-01	0.68 ↓	-0.56	4.25E-02	2.40E-01	0.74 ↓	-0.44
PC(18:0/18:1)	66	788.5214	8.62	1.95E-26	1.46E-23	0.47 ↓	-1.08	1.83E-01	3.81E-01	0.91 ↓	-0.13	6.65E-01	9.66E-01	0.97 ↓	-0.04	1.42E-01	3.85E-01	0.85 ↓	-0.23
PC(18:0/18:2)	99	786.5904	4.76	1.17E-09	1.32E-08	1.54 ↑	0.62	2.98E-01	5.03E-01	0.91 ↓	-0.14	8.13E-01	9.42E-01	1.01 ↑	0.01	4.67E-02	7.82E-01	0.76 ↓	-0.40
PC(18:0/22:6)	138	878.5916	5.01	5.77E-06	4.44E-05	1.33 ↑	0.41	3.55E-01	5.94E-01	0.90 ↓	-0.14	7.71E-01	9.72E-01	0.99 ↓	-0.01	1.11E-01	3.50E-01	0.86 ↓	-0.21
PC(18:1/14:0)	15	732.5392	8.34	9.13E-05	5.38E-04	0.84 ↓	-0.25	7.79E-02	2.21E-01	0.90 ↓	-0.15	2.56E-02	2.12E-01	0.81 ↓	-0.30	4.53E-02	2.46E-01	0.84 ↓	-0.25
PC(18:1/18:1)	55	786.5061	8.63	6.50E-10	9.24E-09	0.61 ↓	-0.72	2.15E-01	4.26E-01	0.89 ↓	-0.16	8.94E-03	1.10E-01	0.64 ↓	-0.65	1.50E-01	3.93E-01	0.77 ↓	-0.37
PC(O-16:0/0:0)	37	504.3061	4.98	5.08E-06	3.00E-05	0.73 ↓	-0.46	5.15E-02	1.45E-01	0.88 ↓	-0.19	4.60E-01	7.34E-01	0.78 ↓	-0.35	4.67E-01	8.99E-01	0.79 ↓	-0.34
PC(O-16:0/18:1)	20	746.558	8.56	1.01E-02	3.01E-02	0.85 ↓	-0.23	6.25E-02	1.69E-01	0.86 ↓	-0.21	3.54E-01	6.70E-01	1.09 ↑	0.13	9.70E-01	9.83E-01	0.94 ↓	-0.09
PC(O-16:0/20:3)	7	770.5673	8.63	6.66E-10	9.96E-09	0.63 ↓	-0.66	6.49E-02	1.69E-01	0.86 ↓	-0.22	6.81E-01	8.68E-01	0.92 ↓	-0.12	3.01E-01	7.54E-01	0.89 ↓	-0.16
PC(O-16:0/20:4)	54	790.5705	8.65	6.14E-07	4.36E-06	1.25 ↑	0.33	8.45E-03	3.44E-02	0.85 ↓	-0.23	3.25E-02	5.45E-01	1.18 ↑	0.23	7.51E-02	8.84E-01	1.15 ↑	0.21
PC(O-16:0/20:4)	54	768.5526	4.37	1.37E-02	3.97E-02	0.91 ↓	-0.14	7.41E-03	3.22E-02	0.85 ↓	-0.23	1.03E-01	5.55E-01	1.18 ↑	0.24	3.15E-01	8.84E-01	1.07 ↑	0.10
PC(O-16:0/20:5)	28	766.5388	8.58	1.00E-04	5.80E-04	1.26 ↑	0.34	9.20E-02	2.45E-01	0.85 ↓	-0.23	8.67E-01	9.73E-01	1.01 ↑	0.01	1.61E-01	4.09E-01	0.88 ↓	-0.18
PC(O-18:0/2:0)	112	590.3227	0.97	1.45E-07	1.18E-06	0.72 ↓	-0.48	1.49E-02	5.52E-02	0.83 ↓	-0.27	2.13E-01	5.55E-01	0.80 ↓	-0.32	3.87E-02	7.32E-01	0.71 ↓	-0.50
PC(P-18:0/18:1)	124	816.5553	4.47	2.89E-04	1.25E-03	1.25 ↑	0.32	4.81E-02	1.32E-01	0.82 ↓	-0.28	5.35E-01	7.95E-01	1.10 ↑	0.13	2.28E-01	7.54E-01	0.87 ↓	-0.21
PC(P-18:0/20:4)	18	794.5454	8.37	9.59E-03	3.45E-02	1.15 ↑	0.20	1.24E-02	6.82E-02	0.82 ↓	-0.29	7.93E-01	9.72E-01	1.02 ↑	0.03	9.61E-01	9.78E-01	1.02 ↑	0.02
PC(P-18:0/22:6)	121	862.5529	4.53	3.66E-05	1.76E-04	0.78 ↓	-0.35	9.70E-03	3.83E-02	0.82 ↓	-0.30	3.88E-01	7.00E-01	1.08 ↑	0.11	3.31E-01	8.84E-01	1.09 ↑	0.13
PE(16:0/18:2)	119	716.5243	4.79	3.90E-01	5.91E-01	0.98 ↓	-0.03	8.47E-03	3.44E-02	0.81 ↓	-0.30	1.63E-01	5.55E-01	1.16 ↑	0.21	1.86E-01	8.84E-01	1.16 ↑	0.21
PE(16:0/20:4)	132	738.5075	4.8	1.72E-04	9.62E-04	0.83 ↓	-0.27	1.98E-03	1.72E-02	0.81 ↓	-0.31	7.60E-01	9.70E-01	1.01 ↑	0.01	2.48E-01	5.07E-01	1.11 ↑	0.16
PE(16:0/22:6)	45	764.5231	8.63	1.01E-04	4.54E-04	0.77 ↓	-0.38	5.03E-03	2.34E-02	0.80 ↓	-0.32	1.93E-01	5.55E-01	1.13 ↑	0.18	2.62E-01	8.84E-01	1.10 ↑	0.13
PG(16:0/22:6)	36	817.5626	8.67	6.33E-06	3.65E-05	0.76 ↓	-0.40	2.34E-03	1.20E-02	0.80 ↓	-0.33	1.56E-01	5.55E-01	1.14 ↑	0.19	2.34E-01	8.84E-01	1.10 ↑	0.14
Phosphocholine	69	184.074	8.37	1.56E-12	3.61E-11	0.65 ↓	-0.61	5.01E-03	3.57E-02	0.79 ↓	-0.34	4.28E-01	8.99E-01	0.89 ↓	-0.16	3.55E-01	6.10E-01	1.11 ↑	0.15
PI(18:0/20:4)	137	885.5502	4.49	2.09E-04	1.14E-03	0.87 ↓	-0.20	3.18E-06	9.77E-05	0.77 ↓	-0.38	2.53E-02	2.12E-01	1.15 ↑	0.20	1.50E-01	3.93E-01	1.10 ↑	0.14
Picolinic acid N-oxide	91	140.0692	0.54	1.65E-06	1.08E-05	0.72 ↓	-0.47	2.05E-03	1.08E-02	0.76 ↓	-0.39	5.57E-01	8.04E-01	0.99 ↓	-0.02	7.86E-01	9.45E-01	0.91 ↓	-0.13
Propionylcarnitine	67	159.0915	0.93	6.83E-03	2.10E-02	1.19 ↑	0.26	4.80E-02	1.37E-01	0.76 ↓	-0.40	4.32E-01	7.20E-01	1.11 ↑	0.16	4.08E-01	8.99E-01	0.91 ↓	-0.13
PS(18:0/18:1)	123	788.5236	4.74	1.68E-12	2.57E-11	0.61 ↓	-0.72	1.02E-03	6.10E-03	0.74 ↓	-0.42	1.79E-02	4.20E-01	0.66 ↓	-0.61	3.81E-01	8.97E-01	0.86 ↓	-0.22
PS(18:0/18:2)	113	810.5996	5.05	2.37E-10	4.01E-09	0.56 ↓	-0.83	3.73E-04	2.59E-03	0.69 ↓	-0.54	3.49E-01	7.55E-01	0.76 ↓	-0.40	6.64E-01	8.61E-01	0.82 ↓	-0.28
SM(d18:1/16:0)	32	747.5158	4.89	3.78E-15	8.31E-14	0.53 ↓	-0.92	3.20E-04	2.25E-03	0.68 ↓	-0.55	8.98E-01	9.71E-01	0.96 ↓	-0.06	3.45E-01	8.84E-01	1.16 ↑	0.22
SM(d18:1/18:0)	24	731.5408	4.17	3.19E-04	1.67E-03	0.85 ↓	-0.23	2.49E-08	2.15E-06	0.68 ↓	-0.55	2.92E-01	8.07E-01	1.17 ↑	0.23	2.70E-02	1.85E-01	1.20 ↑	0.27
SM(d18:1/18:1)	30	729.589	8.47	3.34E-20	4.68E-18	0.55 ↓	-0.87	1.68E-07	9.13E-06	0.66 ↓	-0.59	5.12E-01	9.41E-01	0.91 ↓	-0.13	8.50E-01	9.29E-01	0.99 ↓	-0.01
SM(d18:1/22:0)	92	787.5936	4.76	4.54E-09	5.59E-08	0.77 ↓	-0.39	2.59E-09	4.85E-07	0.66 ↓	-0.61	9.62E-01	9.92E-01	1.03 ↑	0.04	1.17E-01	3.57E-01	1.14 ↑	0.19
Thioetheramide-PC	93	735.5718	4.94	2.79E-11	4.96E-10	0.72 ↓	-0.46	6.11E-09	8.82E-07	0.65 ↓	-0.63	4.54E-01	9.08E-01	1.08 ↑	0.12	7.37E-05	2.59E-03	1.46 ↑	0.55

Thioetheramidephosphatidylcholine	57	757.5559	8.44	1.40E-05	7.36E-05	0.74 ↓	-0.43	4.65E-07	1.05E-05	0.60 ↓	-0.75	1.71E-01	5.55E-01	1.14 ↑	0.19	4.58E-01	8.99E-01	1.07 ↑	0.09
Tyramine	8	121.0846	0.57	4.84E-01	6.60E-01	1.07 ↑	0.09	4.21E-06	5.59E-05	0.58 ↓	-0.78	4.47E-02	5.55E-01	1.28 ↑	0.36	9.08E-01	9.73E-01	0.88 ↓	-0.18
L-Valine	90	118.0873	0.52	1.49E-05	7.75E-05	0.76 ↓	-0.40	3.51E-08	1.17E-06	0.58 ↓	-0.79	2.86E-02	5.19E-01	1.22 ↑	0.29	1.98E-02	4.49E-01	1.21 ↑	0.27
Vitamin E	95	431.3888	4.85	4.26E-03	1.31E-02	0.80 ↓	-0.32	3.91E-06	9.32E-05	0.56 ↓	-0.84	5.06E-02	3.57E-01	1.45 ↑	0.54	1.90E-01	7.10E-01	1.20 ↑	0.27
β-15d-PGJ2	9	329.2104	2.98	1.32E-04	6.15E-04	1.25 ↑	0.33	8.38E-04	4.93E-03	1.39 ↑	0.48	2.72E-01	7.33E-01	0.85 ↓	-0.24	4.76E-01	7.62E-01	0.89 ↓	-0.18

Supplementary.Table3

Group names	Total #	Metabolites
Rad SD 1 effects and Rad SD 4 effects	**77**	Tigecycline
		N-Acetyl-L-phenylalanine
		1,2-Dioleoyl-sn-glycero-3-PC
		20:4 (Cis) PC
		Sphingomyelin
		trans-4-Hydroxy-L-proline
		18:0-22:6 PC
		Palmitoyl sphingomyelin
		14:0 Lyso PC
		Etiocholanolone glucuronide
		16:0-20:4 PC
		Dimethyl glutamate
		C16(Plasm) LPC
		N-(Phenylacetyl)-L-phenylalanine
		18:1-14:0 PC
		16:0-22:6 PC
		L-Valine
		15-Deoxy-.DELTA.12,14-prostaglandin J2 biotinamide
		N-Arachidonylmaleimide
		Tyramine
		18:2 PC (DLPC)
		C16-18:1 PC
		Linoleoylcarnitine
		C18(Plasm)-20:4 PC
		Lyso-PC(16:0)
		Picolinic acid N-oxide
		L-Histidine
		16:0-18:2 PE
		16:1 (.DELTA.9-Cis) PC
		1-Phenoxy-2-propanol
		1,2-Dipalmitoyl-sn-glycero-O-ethyl-3-PC cation
		Sorbitan monostearate
		Oleoyl-L-carnitine
		Leu-Pro
		C18(Plasm)-22:6 PC
		Apiquel
		Arachidonoylthio-PC
		15:0 Lyso PC
		DL-Phenylalanine
		γ-Cholestenol

		Arachidonoyl PAF C-16
		Thioetheramidephosphatidylcholine
		16:0 Dimethyl PE
		L-Alanine, L-isoleucyl-
		Eicosapentaenoyl PAF C-16
		2-Hydroxy-6-methylquinoline-3-carbaldehyde
		1-Docosahexaenoyl-2-stearoyl-sn-glycero-3-phosphocholine
		Mesoporphyrin IX
		11-Deoxy-16,16-dimethylprostaglandin E2
		N-Formylamphetamine
		PC(16:0/16:0)
		17:0 Lyso PC
		4-Amino-2-methylpyrimidine-5-carbonitrile
		18:1 SM (d18:1/18:1)
		Androstenediol
		Palmitoylcarnitine
		K-R-T-L-R-R
		PAF C-18
		C18(Plasm)-18:1 PC
		SM d18:1/22:0
		16:0-18:1 PC
		Dihomo-γ-linolenoyl PAF C-16
		17-Oxobetamethasone
		2,5-Dimethoxy-4-ethylthioamphetamine
		3,6-Dimethyl-4-hydroxycoumarin
		Lyso-PAF C-18
		Acetyl-DL-carnitine
		VAK
		18:0-18:2 PC
		16:0-20:4 PE
		Etiocholanolone glucuronoside
		1-Octadecanoyl-2-octadecenoyl-sn-glycero-3-phosphocholine
		Etiocholan-3α-ol-17-one
		Epitestosterone
		DL-Indole-3-lactic acid
		L-Methionine
		Octanoylcarnitine
**Rad SD 1 only effects**	**33**	18:0 Lyso PC
		Carvedilol
		20:4 PC
		C-16 Ceramide
		2-Methylbutyryl-L-carnitine
		Glabrol
		2-Nitrobenzaldehyde semicarbazone

		N-Acetyl-L-alanine
		Di(2-nonyl) phthalate
		L-Carnitine
		18:1 Lyso PC
		5α-Androstane-3α,17β-diol
		18:0-20:4 PI
		Lyso-sphingomyelin
		Lyso-PAF C-16
		DOM
		(+)-α-Tocopherol
		16:0-22:6 PE
		3-Hydroxybutyrylcarnitine
		16:0-22:6 PG
		Phosphocholine
		Hydroxyprogesterone
		16:0 Lyso PE
		Phe-Phe
		18:0-18:2 PS
		Kynurenic acid
		Methylcarbamyl PAF C-8
		Oleyloxyethylphosphorylcholine
		18:0-18:1 PS
		1,2-Dipalmitoleoyl-sn-glycero-3-phosphocholine
		2-Thio PAF
		14:0-18:0 PC
		Retigabine
**Rad SD 4 only effects**	**2**	PC(16:0/18:0)
		L-Kynurenine

Supplementary Table 4: Pathway analysis as obtained from Mummichog v2.0.6

	Vehicle SD 1 vs. Pre-irradiation		Vehicle SD 4 vs. Pre-irradiation		Ex-Rad I SD 4 vs. Vehicle SD 4		Ex-Rad II SD 4 vs. Vehicle SD 4	
**Pathway**	*overlap size*	*p-value*	*overlap size*	*p-value*	*overlap size*	*p-value*	*overlap size*	*p-value*
**Arachidonic acid metabolism**	-	-	1(1)	3.70E-02	-	-	6(8)	1.68E-04
**Sialic acid metabolism**	5(5)	5.88E-04	-	-	-	-	-	-
**Carnitine shuttle**	13(15)	1.09E-03	-	-	5(15)	2.24E-02	2(9)	4.02E-02
**Linoleate metabolism**	-	-	-	-	5(14)	1.72E-02	3(4)	1.26E-03
**Leukotriene metabolism**	-	-	-	-	2(3)	7.73E-03	3(3)	1.43E-03
**Glycolysis and Gluconeogenesis**	4(4)	1.43E-03	4(4)	1.18E-02	-	-	-	-
**C21-steroid hormone biosynthesis and metabolism**	17(22)	1.76E-03	2(3)	2.38E-02	2(3)	1.25E-02	8(22)	1.22E-02
**Prostaglandin formation from arachidonate**	-	-	-	-	3(6)	4.79E-03	4(6)	3.02E-03
**Glycine, serine, alanine and threonine metabolism**	6(8)	3.02E-03	6(8)	3.38E-02	-	-	1(2)	3.61E-02
**Glycerophospholipid metabolism**	7(12)	1.87E-02	4(8)	2.38E-02	-	-	4(8)	3.36E-03
**Tyrosine metabolism**	7(13)	3.87E-02	3(4)	4.20E-03	-	-	6(11)	3.70E-03
**Urea cycle/amino group metabolism**	4(4)	4.37E-03	5(6)	2.49E-02	-	-	2(4)	4.50E-02
**Glycosphingolipid biosynthesis - globoseries**	3(3)	4.54E-03	1(1)	3.70E-02	-	-	-	-
**Galactose metabolism**	3(3)	4.54E-03	3(3)	3.08E-02	-	-	-	-
**Porphyrin metabolism**	5(6)	4.96E-03	4(5)	2.86E-02	1(1)	3.60E-02	-	-
**Androgen and estrogen biosynthesis and metabolism**	3(4)	3.40E-02	-	-	4(7)	5.63E-03	3(7)	3.38E-02
**Tryptophan metabolism**	-	-	-	-	5(11)	7.65E-03	-	-
**Butanoate metabolism**	-	-	1(1)	3.70E-02	1(1)	4.13E-02	3(4)	8.91E-03
**Glutathione Metabolism**	-	-	-	-	2(3)	1.25E-02	2(3)	4.54E-02
**Purine metabolism**	-	-	-	-	2(3)	1.25E-02	2(3)	4.54E-02
**Vitamin E metabolism**	2(2)	3.98E-02	8(12)	2.13E-02	-	-	-	-
**Ascorbate (Vitamin C) and Aldarate Metabolism**	2(2)	3.98E-02	3(3)	2.14E-02	-	-	-	-
**Limonene and pinene degradation**	-	-	3(3)	2.14E-02	-	-	-	-
**Fatty Acid Metabolism**	-	-	3(3)	2.16E-02	-	-	-	-
**Arginine and Proline Metabolism**	-	-	-	-	2(5)	4.02E-02	3(5)	2.27E-02
**Glycosphingolipid metabolism**	-	-	2(3)	2.38E-02	-	-	-	-
**De novo fatty acid biosynthesis**	-	-	7(10)	2.45E-02	-	-	-	-
**Valine, leucine and isoleucine degradation**	2(2)	3.10E-02	1(1)	3.70E-02	1(1)	4.13E-02	1(1)	2.72E-02
**Fructose and mannose metabolism**	2(2)	2.73E-02	1(1)	3.70E-02	-	-	-	-
**TCA cycle**	2(2)	2.73E-02	-	-	-	-	-	-
**Phosphatidylinositol phosphate metabolism**	-	-	3(3)	3.08E-02	-	-	-	-
**Pyruvate Metabolism**	3(4)	3.42E-02	-	-	-	-	-	-
**Alanine and Aspartate Metabolism**	3(4)	3.42E-02	-	-	1(1)	4.50E-02	-	-
**Vitamin B9 (folate) metabolism**	-	-	-	-	1(1)	3.60E-02	-	-
**N-Glycan Degradation**	-	-	1(1)	3.70E-02	-	-	-	-
**Methionine and cysteine metabolism**	-	-	1(1)	3.70E-02	2(5)	4.02E-02	-	-
**Starch and Sucrose Metabolism**	-	-	1(1)	3.70E-02	-	-	-	-
**Chondroitin sulfate degradation**	-	-	1(1)	3.70E-02	-	-	-	-
**Hexose phosphorylation**	-	-	1(1)	3.70E-02	-	-	-	-
**N-Glycan biosynthesis**	-	-	1(1)	3.70E-02	-	-	-	-
**Squalene and cholesterol biosynthesis**	-	-	1(1)	3.70E-02	-	-	-	-
**Keratan sulfate degradation**	-	-	1(1)	3.70E-02	-	-	-	-
**Caffeine metabolism**	-	-	1(1)	3.70E-02	-	-	-	-
**Biopterin metabolism**	-	-	1(1)	3.70E-02	-	-	-	-
**Heparan sulfate degradation**	-	-	1(1)	3.70E-02	-	-	-	-

**Sphingolipid metabolism**	-	-	1(1)	3.70E-02	-	-	-	-
**Lysine metabolism**	2(2)	3.98E-02	-	-	-	-	-	-
**Bile acid biosynthesis**	-	-	6(9)	4.02E-02	-	-	-	-
**Fatty acid oxidation**	-	-	-	-	1(1)	4.50E-02	-	-
**Fatty acid oxidation, peroxisome**	-	-	-	-	1(1)	4.50E-02	-	-
**Histidine metabolism**	-	-	-	-	-	-	2(3)	4.54E-02
**Beta-Alanine metabolism**	-	-	-	-	-	-	2(3)	4.54E-02
**Vitamin B1 (thiamin) metabolism**	-	-	2(2)	4.71E-02	-	-	-	-
**Xenobiotics metabolism**	-	-	2(2)	4.71E-02	-	-	-	-

Note. Pathway analysis result with positive mode, negative mode, lipidomics profiling, and metabolomics profiling combined.

[a] Numbers in parenthesis indicates the pathway size.
